# Identification and Structure-Activity Studies of 1,3-Dibenzyl-2-aryl imidazolidines as Novel Hsp90 Inhibitors

**DOI:** 10.3390/molecules24112105

**Published:** 2019-06-03

**Authors:** Yajun Liu, Xiaoxia Liu, Lihong Li, Rui Dai, Meiyun Shi, Hongyu Xue, Yong Liu, Hecheng Wang

**Affiliations:** School of Life Science and Medicine, Dalian University of Technology, Dagong Road No. 2, Panjin 124221, China; lxx890720@mail.dlut.edu.cn (X.L.); 1408064107@mail.dlut.edu.cn (L.L.); dairui666@mail.dlut.edu.cn (R.D.); shimy@dlut.edu.cn (M.S.); yliu@dlut.edu.cn (Y.L.); wanghc@dlut.edu.cn (H.W.)

**Keywords:** Hsp90, imidazolidine, anti-cancer, molecule docking, virtual screening

## Abstract

Hsp90 (Heat shock protein 90) is involved in various processes in cancer occurrence and development, and therefore represents a promising drug target for cancer therapy. In this work, a virtual screening strategy was employed, leading to the identification of a series of compounds bearing a scaffold of 1,3-dibenzyl-2-aryl imidazolidine as novel Hsp90 inhibitors. Compound **4a** showed the highest binding affinity to Hsp90α (IC_50_ = 12 nM) in fluorescence polarization (FP) competition assay and the strongest anti-proliferative activity against human breast adenocarcinoma cell line (MCF-7) and human lung epithelial cell line (A549) with IC_50_ values of 21.58 μM and 31.22 μM, respectively. Western blotting assays revealed that these novel Hsp90 inhibitors significantly down-regulated the expression level of Her2, a client protein of Hsp90, resulting in the cytotoxicity of these novel Hsp90 inhibitors. The molecular docking study showed that these novel Hsp90 inhibitors bound to the adenosine triphosphate (ATP) binding site at the N-terminus of Hsp90. Furthermore, structure–activity relationship studies indicated that the *N*-benzyl group is important for the anti-cancer activity of 1,3-dibenzyl-2-aryl imidazolidines.

## 1. Introduction

Hsp90 is a molecular chaperone with the molecular weight of 90 kD in the family of heat shock proteins. It plays an important role in maintaining conformations and biological functions of various oncoproteins, which are indispensable for the occurrence and progression of various cancers, including melanoma, breast cancer, gastrointestinal stromal tumors, and lung cancer [1,2,3,4,5,6]. The expression level of Hsp90 in cancer cells is much higher than it in normal cells, which makes it an attractive drug target for the therapy of cancers [7,8,9,10,11]. Inhibition of Hsp90 in cancer cells causes the degradation of client oncoproteins, and thus Hsp90 inhibitor is considered as a prominent strategy for the therapy of cancers [12,13,14]. In addition to cancer, Hsp90 has been found as potential drug target for neurodegenerative diseases (e.g., Alzheimer’s disease [15], Parkinson disease [16], Huntington disease [17]), viral disease, [18] fungal disease [19], and parasitic disease) [20].

**Figure 1:**
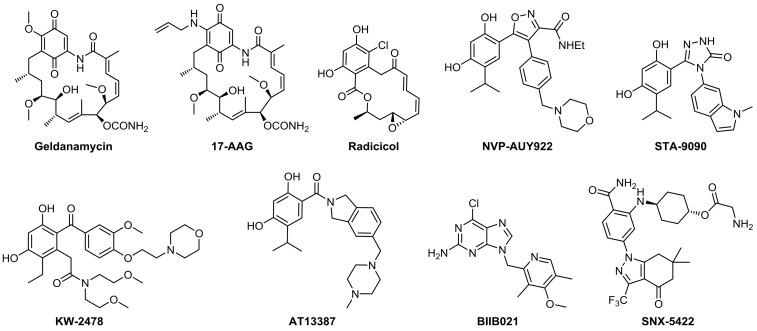
Representative Hsp90 inhibitors in clinical trials.

Binding and hydrolysis of ATP at the N-terminus of Hsp90 provides energy for client protein folding, and inhibiting this process will result in ubiquitinylation of the substrate and induce degradation of client protein via the proteasome. Generally, Hsp90 inhibitors are designed to competitively target the ATP binding site at the N-terminal of Hsp90 and inhibit the ATPase activity of Hsp90 [21,22,23,24]. The first generation of Hsp90 inhibitors were derived from natural products [25]. Geldanamycin is the first identified Hsp90 inhibitor, however the dose and schedule-dependent toxicity limits its further development [26]. Modification of geldanamycin led to the identification of 17-AAG, which was the first Hsp90 inhibitor entered into clinical trials in 1999 [27]. Although it has low toxicity and better water solubility in comparison to geldanamycin, it was recently retired from clinical trials. Radicicol is the second natural Hsp90 inhibitor, but it is inactive in human tumor xenografts in mouse models due to its chemical instability [28]. The following studies developed many small molecule inhibitors with scaffolds of various kinds as the second generation of Hsp90 inhibitors (Figure 1). Inspired by radicicol, many resorcinol-based inhibitors, including NVP-AUY922, [29,30] STA-9090, [31] KW-2478 [32,33], and SNX-5422 [34], have been designed. In addition, purine- [35,36] and benzamide-based [37] small molecule Hsp90 inhibitors have also been developed for clinical trials. 

There are eighteen Hsp90 inhibitors that have been entered into clinical trials. Unfortunately, no Hsp90 inhibitors have been approved by the Food and Drug Administration (FDA). Thus, there is still a need to further develop and amplify inhibitor scaffolds for more novel Hsp90 inhibitors. Herein, we described a virtual screening-based method for identifying novel Hsp90 inhibitors. A virtual library containing 300,000 small molecules was screened using Discovery Studio, and 1,3-dibenzyl-2-aryl imidazolidines were identified as novel Hsp90 inhibitors in biochemical and cellular assays.

## 2. Results and Discussion

### 2.1. Structure Based Virtual Screening

**Figure 2:**
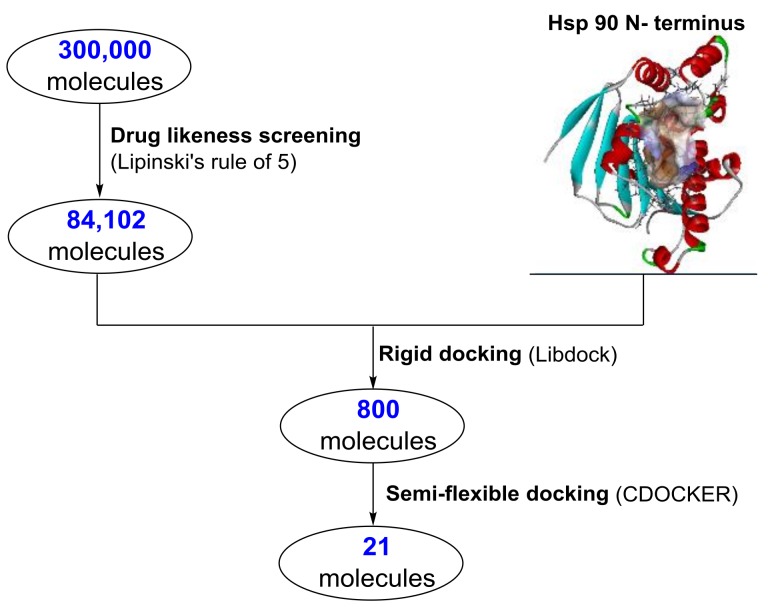
Workflow of the virtual screening protocol for Hsp90 inhibitor development.

In order to obtain Hsp90 inhibitors with novel central scaffolds, a small molecule library containing 300,000 molecules was downloaded from Specs and virtually screened. As shown in Figure 2, this small molecule library was initially screened by Lipinski’s Five Rules to exclude molecules with possibly poor drug-like properties. In total, 84,102 molecules entered the next round for molecule docking. The ATP binding site at the N-terminal of Hsp90 (protein data bank code: 4CE1) was chosen as the active site. Molecule docking was performed by 2 steps: (1) rigid docking module LibDock provided fast screening and the molecules were ranked by LibDock score; (2) the top 800 molecules were further docked with Hsp90 using a semi-flexible docking method CDOCKER and ranked by “CDOCKER_ENERGY”. Finally, the top 21 molecules were selected for further biological studies.

### 2.2. Biological Evaluation of Molecules Identified from Virtual Screening

**Figure 3:**
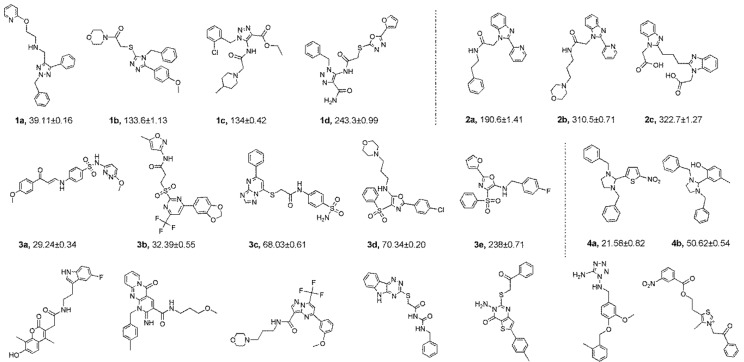
Chemical structures and anti-proliferative activity (IC_50_ = the mean ± SD μM, *n* = 3) against MCF-7 cells of the top 21 molecules identified from the virtual screening (positive control: 17-AAG, IC_50_ = 7.18 ± 0.13 μM).

The 21 compounds were divided into 5 groups by their different characteristic scaffolds, including triazoles (**1a**–**1d**), benzimidazoles (**2a**–**2c**), aryl sulfonyls (**3a**–**3e**), imidazolidines (**4a**,**4b**), and others (**5a**–**5g**) (Figure 3). The anti-proliferative activity against MCF-7 cells of these 21 compounds were evaluated using MTT assay. The IC_50_ values ranged from 21.58 μM to 322 μM. Compounds **4a** and **4b**, each bearing an imidazolidine scaffold, attracted our attention because of their relatively strong anti-cancer activity and novel chemical structure. Compound **4a** exhibited the strongest anti-proliferative activity towards MCF-7 cells, with an IC_50_ value of 21.58 μM. To the best of our knowledge, 2-aryl-imidazolidines have never been reported as either anti-cancer agents or Hsp90 inhibitors. Thus, **4a** was taken as a hit compound to develop more novel Hsp90 inhibitors with 2-aryl imidazolidine as the central scaffold.

### 2.3. Molecule Docking Analysis of Hsp90-***4a*** Complex

**Figure 4:**
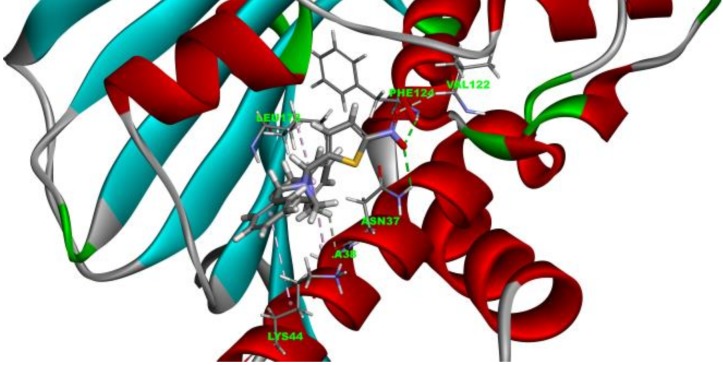
Molecular docking analysis of the **4a**-yeast Hsp90 complex. Predicted binding mode of **4a** and Hsp90. Hydrogen bonds are indicated by green dashed lines. The Pi-alkyl interaction is shown by a pink dashed line.

To gain a better understanding of the binding mode of **4a** and Hsp90, the molecular docking result of **4a** with the N-terminal of the ATP binding pocket of the yeast Hsp90 was studied. As shown in Figure 4, **4a** occupied the ATP binding cavity at the N-terminal of Hsp90. The nitro group on the thiophene ring formed 2 hydrogen bonds with PHE124 and ASN37, respectively. Benzyl groups formed hydrophobic bonds with amino acid residues of Hsp90. This result indicated that a hydrogen bond acceptor at the 2-position of imidazolidine and a hydrophobic fragment at the nitrogen atoms are favorable for this kind of molecule to bind Hsp90.

### 2.4. Structure-Activity Relationship (SAR) Studies

In order to get more Hsp90 inhibitors with potent anti-cancer activities, a series of 1,3-dibenzyl-2-aryl imidazolidines with different aryl groups (**4c**–**4r**) were designed based on the predicted binding mode of **4a** and Hsp90. As Table 1 showed, these kinds of compounds were readily synthesized through a condensation of *N,N*′-dibenzyl ethylenediamine with the corresponding (hetero)aryl aldehydes. Pure products were easily obtained by filtration and washing with cold ethanol. The nuclear magnetic resonance (NMR) spectra of all synthesized compounds can be found in the Supplementary Materials. Anti-cancer activities of the synthesized compounds were evaluated using human breast cancer MCF-7 cell line and adenocarcinomic human alveolar basal epithelial A549 cell line. 17-AAG was used as the positive control. 

As shown in the molecule docking study, the nitro group played an important role in the interaction of **4a** and Hsp90, and we studied the effect of the nitro group on antitumor activity. Removal of the nitro group (**4c**) or replacing nitro with other functional groups (**4d**–**4f**) led to a decrease in the cytotoxicity. When the thiophene ring in **4a** was replaced by the phenyl ring, the position effect of the nitro group was observed as *ortho*-nitro-substituted phenyl (**4g**), which gave better activity than the *para*-substituted one (**4h**). Considering that nitrothiophene is a hydrogen bond acceptor, various analogues (**4i**–**4r**) bearing a broad range of hydrogen bond acceptors were synthesized. Among these synthesized analogues, **4i** bearing a 2-fuloro-5-chloro ring exhibited the strongest anti-proliferative activity against MCF-7 cells with IC_50_ of 27.86 μM, and **4j** with a 2-chloro-thiazol-5-yl group inhibited the proliferation of A549 cells with IC_50_ of 35.3 μM. 

The effect of different substituents on two nitrogen atoms on the biological activity was subsequently studied (Table 2). The benzyl group is more favorable as the anticancer activity significantly decreased when the benzyl group of a larger size was replaced by smaller groups, including phenyl (**6a**–**6d**), ethyl (**7a**, **7b**), and methyl groups (**8a**). This may be because small groups cannot form stable hydrophobic bonds with amino acid residues in the ATP binding site of Hsp90.

### 2.5. Binding Affinity Assay

In order to invalidate the inhibitory effect of these imidazolidines on Hsp90, 8 compounds were selected for Fluorescence polarization (FP) competitive binding assay. As shown in Table 3, 6 of the 8 compounds in this series exhibited FP IC_50_ values at the nanomolar level, indicating high binding affinity to Hsp90α. Consistent with the anti-proliferative activity, **4a** showed the strongest binding affinity to human Hsp90α with IC_50_ value of 12 nM in the FP assay, compared with other analogues.

### 2.6. Western Blotting Assay

**Figure 5:**
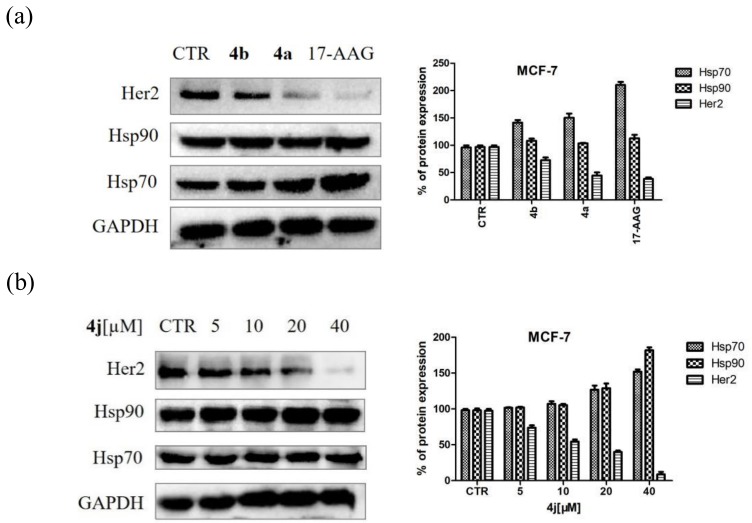
Western blotting assay. (**a**) Effects of 17-AAG, **4a**, and **4b** on the expression of Her2, Hsp70, and Hsp90 in the MCF-7 cell line. Glyceraldehyde 3-phosphate dehydrogenase (GAPDH) was used in a loading control. (**b**) Effects of compound **4j** (0, 5, 10, 20, and 40 µM) on the expression of Her2, Hsp70, and Hsp90 in the MCF-7 cell line.

In order to get more evidence of the Hsp90 inhibition ability of 1,3-dibenzyl-2-aryl imidazolidines, the effects of **4a** and **4b** on the expression level of Her2, an important client protein of Hsp90, was studied. The expression levels of Her2, Hsp70, and Hsp90 were measured by western blotting, using 17-AAG as the positive control. MCF-7 cells were incubated with compound **4a** and **4b** at IC_50_ concentrations for 24 h (Figure 5a). Compound **4a** and **4b** significantly down-regulated the expression level of Her2. It is interesting that **4a** at IC_50_ concentration down-regulated the expression level of Her2 to 48%, indicating that the anti-proliferative activity of **4a** against MCF-7 cells was a consequence of Hsp90 inhibition. On the other side, **4a** and **4b** significantly induced the expression of Hsp70 and slightly increased the expression of Hsp90. In another experiment, after exposure for 24 h to **4j** at different concentrations, the expression levels of Her2, Hsp70, and Hsp90 in MCF-7 cells were regulated by **4j** in a concentration-dependent manner (Figure 5b). Taken together, 1,3-dibenzyl-2-aryl imidazolidines exhibited their anticancer activity through modulating the Hsp90 function.

## 3. Materials and Methods

### 3.1. Virtual Screening

The small molecule library for virtual screening was downloaded from Specs (www.specs.net). Virtual screening was carried out using Discovery Studio 2016 (Accelrys^®^, San Diego, CA, USA). The small library was first filtered by Lipinski’s rule of five (“Molecule Weight” was set to “200–500”, “H_Acceptors” to “less than 10”, “H_Donors” to “less than 5”, “ClogP” to “less than 5”). The resulting molecules were further screened by molecular docking. The cocrystal structure of radicicol bound to yeast Hsp90 (PDB ID: 4CE1; 2.01 Å resolution) was downloaded from the Protein Data Bank (www.rcsb.org). Radicicol was removed from the complex and the resulting protein was prepared using “prepare protein” to remove water molecules and add hydrogens. The binding site of radicicol in Hsp90 was selected as the active site (radius: 9 Å). The small molecule library was imported and docked with Hsp90 using CHARMm (Chemistry at HARvard Macromolecular Mechanics) force field. The molecule docking was first carried out using “LibDock” and ranked by “LibDockScore”. The top 1% of ranked molecules were further docked into the same active site of Hsp90 using “CDOCKER” and ranked by “-CDOCKER_ENERGY”. The predicted binding mode of molecule **4a** was selected according to the lowest binding free energy.

### 3.2. Evaluation of Anti-proliferative Activity In Vitro

The anti-proliferative activity of compounds was determined by MTT (3-[4,5-dimethylthiazole-2-yl]-2,5-diphenyltetrazolium bromide) assay on MCF-7 and A549 cell lines, respectively. Cells were purchased from the Institute of Basic Medical Science, Chinese Academy of Medical Science (Beijing, China). Cells were maintained in RPMI-1640 with 10% fetal bovine serum (FBS) and 1% Penicillin-Streptomycin Solution (PS) and cultured at 37 °C in a humidified atmosphere containing 5% CO_2_. Compounds to be evaluated were dissolved in DMSO (dimethyl sulfoxide), diluted in RPMI-1640, and stored at −20 °C before use. Cells were seeded in 96-well plates at a density of 5000 cells/well (200 μL) and incubated overnight. Culture medium was replaced with fresh RPMI (Roswell Park Memorial Institute)-1640 containing compounds in different concentrations (100 μM–50 μM–25 μM–12.5 μM–6.25 μM–3.125 μM). Then, cells were incubated in a 5% CO_2_ incubator for 48 h. The 20 μL MTT solution (5 mg/mL) was added to each well and incubated at 37 °C. After 4 h, the medium was removed and formazan crystals were dissolved in 100 μL DMSO. Absorbance was measured at a wavelength of 490 nm using Synergy H1 (BioTek Instruments, Inc., Winooski, VT, USA). IC_50_ values were calculated by GraphPad Prism 5 (GraphPad Software, Inc., San Diego, CA, USA). 

### 3.3. Fluorescence Polarization (FP) Enzymatic Assay

In vitro inhibitory activity of compounds on Hsp90 was carried out in 96-well plates by FP assay using Hsp90α N-Terminal Domain Assay Kit (BPS Bioscience, Catalog #50293, CA, USA) according to the manufacturer’s instructions. In brief, the Hsp90 assay buffer (15 μL), 40 mM dithiothreitol (DTT) (5 μL), 2 mg/mL BSA (5 μL), and H_2_O (40 μL) were added to the plate. To each well, 100 nM fluorescein isothiocyanate (FITC)-labeled geldanamycin (5 μL) and diluted compounds in different concentrations (0.5, 5, 50, 500 μM, 10 μL) were added in an orderly manner. The reactions were initiated by adding 20 μL of Hsp90α (17 ng/μL). After 2.5 h incubation with slow shaking at room temperature, the fluorescence intensity was measured at λex 485 nm and λem 530 nm using Synergy H1 (BioTek Instruments, Inc.). The data were analyzed using GraphPad Prism 5 (GraphPad Software, Inc.).

### 3.4. Western Immune Blotting Assays

Primary antibodies against Hsp70, Hsp90, Her2, and GAPDH were bought from Abcam (Cambridge, MA, USA). HRP (horseradish peroxidase) labeled anti-rabbit immunoglobulin G (H + L) secondary antibody was purchased from Abbkine (Redlands, CA, USA). MCF-7 cells were cultured as described above to a density of 10^6^ cells per dish. The cells were treated with different concentrations of drugs or 17-AAG in RPMI-1640 for 24 h at 37 °C. Cells were washed with cold phosphate buffered saline (PBS, pH = 7.4) three times and lysed in radio immunoprecipitation assay (RIPA) lysate (containing 1% phenylmethanesulfonyl fluoride (PMSF)) on ice for 45 min. The lysates were collected and clarified at 12,000× *g* for 20 min at 4 °C using high speed refrigerated centrifuge. Protein concentration was determined by the bicinchoninic acid (BCA) protein assay kit. The protein sample (20 µg) was electrophoresed using 8% SDS-PAGE (sodium dodecyl sulfate- polyacrylamide gel electrophoresis), transferred to poly(vinylidene fluoride) (PVDF) membranes, and then blocked for 1 h in 5% skim milk in TBST (20 mM Tris-HCl pH 7.4, 100 mM NaCl, and 0.1% Tween 20). The membranes were immunoblotted with primary antibodies for 2 h at room temperature. After incubation with an HRP anti-rabbit IgG (H + L) (1:100,000) as a secondary antibody, the bands were detected using the ECL^TM^ Prime Western Blotting Detection System (ProteinSimple, San Jose, CA, USA). The density of proteins was determined using the AlphaView SA (Alpha Innotech Corp., version 3.4.0.0, San Leandro, CA, USA).

### 3.5. Chemistry

#### 3.5.1. General Information

All chemicals were purchased as reagent grade and used without further purification. The ^1^H and ^13^C-NMR spectra were carried out on an AVANCE III HD 500 MHz nuclear magnetic resonance spectrometer (Bruker, Billerica, MA, USA). The high resolution mass spectrometry (HRMS) was carried out on a Q Exactive mass spectrometer (Thermo Fisher, Waltham, MA, USA) with electrospray ionization ESI) as the ionization source.

#### 3.5.2. General Procedure for the Preparation of 1,3-Dibenzyl-2-aryl Imidazolidine **4c**–**4r**

The corresponding aldehydes (1.0 mmol) were added to a solution of *N,N*′-dibenzyl ethylenediamine (480 mg, 2.0 mmol) in aqueous ethanol (50%, 3 mL). The reaction mixture was stirred at room temperature until the complete consumption of aldehydes, as determined by thin layer chromatography (TLC). The mixture was filtered and the filter cake was washed with a small amount of water and cold ethanol to afford the pure product.

#### 3.5.3. General Procedure for the Preparation of *N,N*′-Diphenyl-2-aryl Imidazolidine **6a**–**6d**

The corresponding aldehyde (1.0 mmol) was added to a solution of *N,N*′-diphenyl ethylenediamine (424 mg, 2.0 mmol) in aqueous ethanol (50%, 3 mL). The reaction mixture was stirred at room temperature until the complete consumption of aldehydes, as determined by TLC. The mixture was filtered and the filter cake was washed with a small amount of water and cold ethanol to afford the pure product.

#### 3.5.4. General Procedure for Preparation of 1,3-Diethyl-2-aryl Imidazolidine **7a**,**7b**

The corresponding aldehyde (1.0 mmol) was added to a solution of *N,N*′-diethyl-ethylenediamine (430 µL, 3.0 mmol) in aqueous ethanol (50 %, 3 mL). The reaction mixture was stirred at 80 °C until the complete consumption of aldehydes, as determined by TLC. After cooling to room temperature, the reaction mixture was extracted with ethyl acetate and water. The organic layer was dried over anhydrous MgSO_4_ and concentrated in vacuum to afford the corresponding product.

#### 3.5.5. General Procedure for Preparation of *N*,*N*′-Dimethyl-2-aryl Imidazolidine **8a**

The corresponding aldehyde (1.0 mmol) was added to a solution of *N,N*′-dimethyl-ethylenediamine (323 µL, 3.0 mmol) in aqueous ethanol (50%, 3 mL). The reaction mixture was stirred at 80 °C for 3 h until the complete consumption of aldehydes, as determined by TLC. After cooling to room temperature, the reaction mixture was extracted with ethyl acetate and water. The organic layer was dried over anhydrous MgSO_4_ and concentrated in a vacuum to afford the corresponding product.

## 4. Conclusions

In this work, a series of novel Hsp90 inhibitors with a scaffold of 1,3-dibenzyl-2-aryl imidazolidine were identified using a strategy of virtual screening. These novel Hsp90 inhibitors showed evident anti-proliferative activity against MCF-7 and A549 cell lines. The compounds of this kind exhibited strong binding affinity to human Hsp90α in FP assay. Western immune blotting assays revealed that these compounds displayed the characteristic molecular biomarker of Hsp90 inhibition, such as the downregulation of Her2 and upregulation of Hsp70 and Hsp90 in MCF-7 cell lines. Typically, **4a** at IC_50_ concentration down-regulated the expression level of Her2 to 48%, providing an additional experimental evidence that **4a** exhibited its anti-proliferative activity by inhibiting the Hsp90 chaperone. Molecule docking studies revealed the aryl moiety is responsible for the formation of hydrogen bonds and benzyl moieties participate in the hydrophobic bond formation with amino acid residues in the ATP binding pocket at the N-terminal of Hsp90.

In conclusion, our work provided **4a** as a novel and promising lead for further development of Hsp90 inhibitors as anticancer drugs. It should be noted that these imidazolidines, such as **4a**, exhibited strong Hsp90 inhibition ability in FP assay, but the IC_50_ values of **4a** in cellular antiproliferation assay are at the micromole level. This discrepancy may be attributed to the poor cellular uptake efficacy or instability of the test compound. Our future work will focus on the in vitro pharmacokinetic evaluation of **4a**, and a structure-activity study on the imidazolidine ring by replacing it with a range of bioisosteres.

## 5. Patents

Part of the work reported in this paper has been applied for a Chinese patent (Y. Liu, X. Liu, M. Shi, H. Wang, CN108822089A, 2018).

## Figures and Tables

**Table 1:**
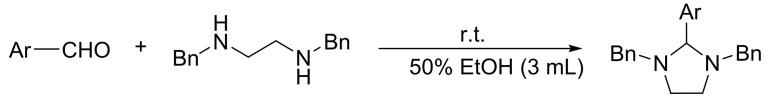
Structure of **4a**–**4r** and their IC_50_ values (μM) on MCF-7 and A549 cell lines, 48-h post treatment.

Entry	Product	Ar	IC_50_^a^ (μM)	
			MCF-7	A549
1	4a	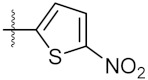	21.58 ± 0.57	31.22 ± 0.31
2	4b	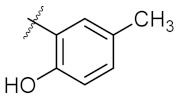	50.62 ± 0.26	88.58 ± 0.08
3	4c	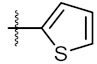	80.04 ± 0.63	78.18 ± 0.15
4	4d	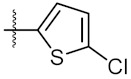	68.35 ± 0.44	56.30 ± 1.27
5	4e	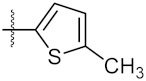	119.30 ± 0.42	112.80 ± 1.41
6	4f	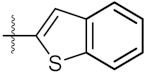	219.40 ± 0.57	135.80 ± 1.41
7	4g	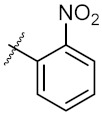	40.91 ± 0.05	40.64 ± 0.51
8	4h	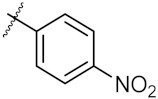	117.70 ± 0.01	55.60 ± 0.49
9	4i	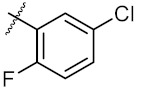	27.86 ± 0.01	59.27 ± 0.83
10	4j	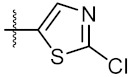	33.12 ± 0.23	35.30 ± 0.14
11	4k	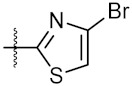	77.01 ± 0.01	498.80 ± 0.25
12	4l	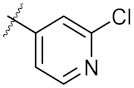	31.48 ± 0.60	64.66 ± 0.48
13	4m	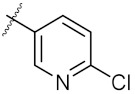	76.42 ± 0.31	70.42 ± 0.10
14	4n	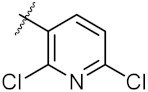	40.36 ± 0.51	52.11 ± 0.50
15	4o	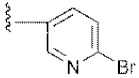	53.12 ± 0.77	56.92 ± 1.27
16	4p	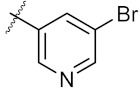	45.25 ± 0.63	72.87 ± 0.66
17	4q	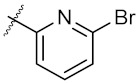	46.97 ± 0.04	71.36 ± 0.88
18	4r	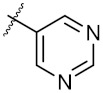	51.37 ± 0.54	80.78 ± 1.00
19	17-AAG	-	7.18 ± 0.13	3.54 ± 0.08

^a^ The IC_50_ values are shown as the mean ± SD (μM) from three separate experiments.

**Table 2:**
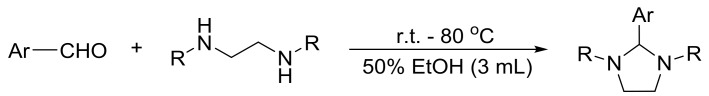
Effect of substituents of nitrogen atoms on the anti-proliferative activity against MCF-7 cells and A549 cells, respectively.

Entry	Product	R	Ar	IC_50_^a^ (μM)	
				MCF-7	A549
1	**6a**	Ph	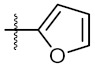	40.77 ± 0.39	123.40 ± 0.85
2	**6b**	Ph	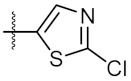	161.60 ± 0.57	172.00 ± 1.41
3	**6c**	Ph	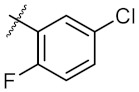	43.67 ± 1.03	46.14 ± 0.20
4	**6d**	Ph	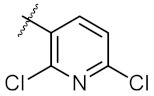	323.00 ± 2.28	248.60 ± 0.57
5	**7a**	Et	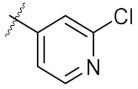	>500	250.10 ± 1.27
6	**7b**	Et	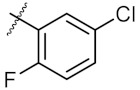	158.90 ± 0.14	164.00 ± 1.41
7	**8a**	Me	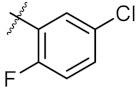	250.10 ± 0.27	148.70 ± 1.56

^a^ The IC_50_ values are shown as the mean ± SD (μM) from three separate experiments.

**Table 3:** FP activities on Hsp90 of selected 2-aryl imidazolidines.

Compounds	FP IC_50_ (μM) ^a^
17-AAG	0.017 ± 0.003
**4a**	0.012 ± 0.010
**4b**	0.612 ± 0.014
**4d**	0.672 ± 0.010
**4i**	0.610 ± 0.014
**4j**	0.310 ± 0.014
**4n**	0.102 ± 0.010
**6c**	1.930 ± 0.013

^a^ The IC_50_ values are shown as the mean ± SD (μM) from three separate experiments.

## Supplementary Materials

The following are available online, Analytical data and NMR spectra of prepared compounds.
