# Supplementary material for: Identification and Structure-Activity Studies of 1,3-Dibenzyl-2-aryl imidazolidines as Novel Hsp90 Inhibitors
Source: Molecules. 2019 Jun 3;24(11):2105. doi: 10.3390/molecules24112105 (PMC6600241; doi:10.3390/molecules24112105)
Supplement: Supplementary file 1 [file molecules-24-02105-s001.pdf]

Communication

Identification and Structure-Activity Studies of 1,3-Dibenzyl-2-Aryl imidazolidines as Novel Hsp90 Inhibitors

Yajun Liu *, Xiaoxia Liu, Lihong Li, Rui Dai, Meiyun Shi, Hongyu Xue *, Yong Liu and Hecheng Wang

[1] School of Life Science and Medicine, Dalian University of Technology, Dagong Road No. 2, Panjin, 124221, China; yjliu85@dlut.edu.cn

* Correspondence: yjliu85@dlut.edu.cn (Y.L.), hongyuxue@dlut.edu.cn(H.X.); Tel.: +86-427-263-1427

Supplementary Material

1. Analytical data of prepared compounds

1,3-dibenzyl-2-thiophene-imidazolidine (**4c,** CAS No. 311788-17-7)[1]. White solid, 167 mg, yield 50%. ^{1}H NMR (500 MHz, $CDCl_3$) :δ (ppm) 7.37 (d, *J* = 5.0 Hz, 1H), 7.31-7.24 (m, 8H), 7.20 (t, *J* = 7.0 Hz, 2H), 7.14 (d, *J* = 3.5 Hz, 1H), 6.95 (m, 1H), 4.25 (s, 1H), 3.93 (d, *J* = 13.0 Hz, 2H), 3.26 (d, *J* = 13.0 Hz, 2H), 3.15-3.13 (m, 2H), 2.52-2.50 (m, 2H); ^{13}C NMR (126 MHz, $CDCl_3$) :δ 146.0, 138.9, 128.6, 128.1, 127.5, 126.8 , 126.6, 125.7 , 83.5 , 56.8, 50.3.

1,3-dibenzyl-2-(5-chlorothiophene)-imidazolidine(**4d**). White solid, 221 mg, yield 60%. ^{1}H NMR (500 MHz, $CDCl_3$) :δ (ppm) 7.45-7.15 (m, 10H), 6.87 (d, *J* = 3.5 Hz, 1H), 6.74 (d, *J* = 3.5 Hz, 1H), 4.16 (s, 1H), 3.94 (d, *J* = 13.0 Hz, 2H), 3.29 (d, *J* = 13.0 Hz, 2H), 3.21-3.04 (m, 2H), 2.59-2.43 (m, 2H);^{13}C NMR (126 MHz, $CDCl_3$) :δ 145.3, 138.7, 131.2, 128.5, 128.2, 126.9, 126.6, 124.9, 83.7, 56.7, 50.2;HRMS (ESI, m/z) for $C_{21}H_{22}ClN_2S$ calcd, 369.1192 $[M+H]^+$; found, 369.1179 $[M+H]^+$.

1,3-dibenzyl-2-(5-Methylthiophene)-imidazolidine(**4e**). Yellow solid, 209 mg, yield 60%. ^{1}H NMR (500 MHz, $CDCl_3$) :δ (ppm) δ 7.37-7.17 (m, 10H), 6.93 (d, *J* = 3.4 Hz, 1H), 6.68-6.49 (m, 1H), 4.13 (s, 1H), 3.96 (d, *J* = 13.0 Hz, 2H), 3.23 (d, *J* = 13.0 Hz, 2H), 3.20-3.06 (m, 2H), 2.52-2.49 (m, 3H), 2.49-2.36 (m, 2H);^{13}C NMR (126 MHz, $CDCl_3$) :δ 143.2, 141.1, 139.1, 128.6, 128.1, 127.5, 126.8, 123.8, 83.9, 56.8, 50.2, 15.7;HRMS (ESI, m/z) for $C_{22}H_{25}N_2S$ calcd, 349.1738 $[M+H]^+$; found, 349.1723 $[M+H]^+$.

1,3-dibenzyl-2-(1-benzothiophene)-imidazolidine(**4f**). White solid, 269 mg, yield 70%. ^{1}H NMR (500 MHz, $CDCl_3$) :δ (ppm) 7.87 (dd, J = 8.5, 4.5 Hz, 1H), 7.85-7.66 (m, 1H), 7.50-7.04 (m, 13H), 4.34 (s, 1H), 3.99 (d, *J* = 13.0 Hz, 2H), 3.32 (d, *J* = 13.0 Hz, 2H), 3.28-3.10 (m, 2H), 2.67-2.48 (m, 2H); ^{13}C NMR (126 MHz, $CDCl_3$) :δ 147.6, 140.7, 139.1, 138.8, 128.6, 128.1, 126.9, 124.31, 124.30, 123.8, 123.3, 122.8, 83.9, 58.1, 50.4; HRMS (ESI, m/z) for $C_{25}H_{25}N_2S$ calcd, 385.1738 $[M+H]^+$; found, 385.1732 $[M+H]^+$.

1,3-dibenzyl-2-(2-nitro-phenyl)- imidazolidine (**4g,** CAS No. 303142-02-1) [2]. Yellow solid, 112 mg, yield 30%. ^{1}H NMR (500 MHz, $CDCl_3$) :δ (ppm) 8.23 (d, *J* = 8.0 Hz, 1H), 7.63 (t, *J* = 7.5 Hz, 2H), 7.40 (td, *J* = 8.0, 1.0 Hz, 1H), 7.27-7.15 (m, 10H), 4.65 (s, 1H), 3.81 (d, *J* = 13.0 Hz, 2H), 3.44 (d, *J* = 13.0 Hz, 2H), 3.20- 2.96 (m, 2H), 2.69-2.43 (m, 2H); ^{13}C NMR (126 MHz, $CDCl_3$)： δ 151.8, 139.0, 136.0, 132.2, 131.7, 128.8, 128.3, 128.1, 126.9, 122.7, 82.1, 57.4, 50.8.

1,3-dibenzyl-2-(4-nitro-phenyl)- imidazolidine (**4h,** CAS No. 304668-59-5)[2]. Light yellow solid, 127 mg, yield 34%. ^{1}H NMR (500 MHz, $CDCl_3$) :δ (ppm) 8.39 (s, 1H), 8.21 (d, *J* = 9.0 Hz, 2H), 8.08 (d, *J* = 9.0 Hz, 1H), 7.75 (d, *J* = 9.0 Hz, 2H), 7.24-7.18 (m, 8H), 4.00 (s, 1H), 3.71 (d, *J* = 13.0 Hz, 2H), 3.33 (d, *J* = 13.0 Hz, 2H), 3.22 (d, *J* = 4.0 Hz, 2H), 2.59 (d, *J* = 4.0 Hz, 2H); ^{13}C NMR (126 MHz, $CDCl_3$) :δ 148.9, 148.1, 138.5, 130.5, 130.2, 128.4, 128.2, 127.0, 124.3, 123.3, 87.7, 57.0, 50.9.

1,3-dibenzyl-2-(5-chloro-2-fluoro-phenyl)-imidazolidine (**4i**). White solid, 160 mg, yield 42%. ^{1}H NMR (500 MHz, $CDCl_3$) :δ (ppm) 7.95 (dd, *J* = 6.0, 3.0 Hz, 1H), 7.32-7.19 (m, 11H), 6.95 (t, *J* = 9.5 Hz, 1H), 4.42 (s, 1H), 3.80 (d, *J* = 13.0 Hz, 2H), 3.39 (d, *J* = 13.0 Hz, 2H), 3.28-3.11 (m, 2H), 2.58 (q, *J* = 4.5 Hz, 2H); ^{13}C NMR (126 MHz, $CDCl_3$) :δ 160.9 (d, *J* = 246 Hz), 138.7, 130.4 (d, *J* = 4.5 Hz), 129.7 (d, *J* = 5.9 Hz),129.65 (d, *J* = 15.1 Hz) 129.61 (d, *J* = 8.6 Hz), 128.5, 128.1, 126.9, 116.3 (d, *J* = 24 Hz), 79.8, 57.0, 50.7; HRMS (ESI, m/z) for $C_{23}H_{23}ClFN_2$ calcd, 381.1534$[M+H]^+$; found, 381.1520 $[M+H]^+$.

1,3-dibenzyl-2-(2-chloro-5-thiazolyl)-imidazolidine (**4j**). White solid, 77 mg, yield 21%. ^{1}H NMR (500 MHz, $CDCl_3$) :δ (ppm) 7.48 (d, *J* = 11.5 Hz, 1H), 7.30-7.27 (m, 7H), 7.2-7.21 (m, 3H), 4.22 (s, 1H), 3.93 (d, *J* = 13.0 Hz, 2H), 3.35 (d, *J* = 13.0 Hz, 2H), 3.16-3.08 (m, 2H), 2.59-2.51 (m, 2H); ^{13}C NMR (126 MHz, $CDCl_3$): δ 153.6, 144.5, 140.5, 138.2, 128.4, 128.3, 127.1, 81.2, 56.7, 50.4; HRMS (ESI, m/z) for $C_{20}H_{21}ClN_3S$ calcd, 370.1145 $[M+H]^+$; found, 370.1139 $[M+H]^+$.

1,3-dibenzyl-2-(3-bromo-5-thiazolyl)-imidazolidine (**4k**). Light pink solid, 132 mg, yield 32%. ^{1}H NMR (500 MHz, $CDCl_3$) :δ (ppm) 7.30-7.26 (m, 9H), 7.24-7.22 (m, 2H), 4.52 (s, 1H), 3.95 (d, *J* = 12.0 Hz, 2H), 3.56 (d, *J* =13.0 Hz, 2H), 3.13 (d, *J* = 3.5 Hz, 2H), 2.62 (d, *J* = 3.5 Hz, 2H); ^{13}C NMR (126 MHz, $CDCl_3$) :δ 175.6,

138.3, 128.5, 128.2, 127.1, 123.8, 118.8, 83.1, 56.8, 50.5; HRMS (ESI, m/z) for $C_{20}H_{21}BrN_3S$ calcd, 414.0640 [M+H]$^+$; found, 414.0649 [M+H]$^+$.

1,3-dibenzyl-2-(3-chloro-4-pyridyl)-imidazolidine (**4l**). White solid, 164 mg, yield 45%. ^{1}H NMR (500 MHz, $CDCl_3$): δ (ppm) 8.35 (d, *J* = 5.0 Hz, 1H), 7.47 (s, 1H), 7.41 (d, *J* = 5.0 Hz, 1H), 7.27 (s, 1H), 7.26-7.18 (m, 9H), 3.91 (s, 1H), 3.72 (d, *J* = 13.0 Hz, 2H), 3.38 (d, *J* = 13.0 Hz, 2H), 3.19 (q, *J* = 5.0 Hz, 2H), 2.60 (q, *J* = 5.0 Hz, 2H); ^{13}C NMR (126 MHz, $CDCl_3$) :δ 154.4, 151.5, 149.5, 138.3, 128.5, 128.2, 127.2, 124.8, 122.9, 86.9, 57.2, 51.0; HRMS (ESI, m/z) for $C_{22}H_{23}ClN_3$ calcd, 364.1580 [M+H]$^+$; found, 364.1509 [M+H]$^+$.

1,3-dibenzyl-2-(4-chloro-3-pyridyl)-imidazolidine (**4m**). White solid, 178 mg, yield 49%. ^{1}H NMR (500 MHz, $CDCl_3$) :δ (ppm) 8.46 (d, *J* = 1.5 Hz, 1H), 7.99 (dd, *J* = 10.0, 2.0 Hz, 1H), 7.36 (d, *J* = 8.0 Hz, 1H), 7.29-7.26 (m, 4H), 7.22 (d, *J* = 7.5 Hz, 6H), 3.93 (s, 1H), 3.74 (d, *J* = 13.0 Hz, 2H), 3.34 (d, *J* = 13.0 Hz, 2H), 3.22 (q, *J* = 4.5 Hz, 2H), 2.59 (q, *J* = 4.5 Hz, 2H); ^{13}C NMR (126 MHz, $CDCl_3$) :δ 151.6, 150.7, 139.8, 138.4, 135.7, 128.4, 128.2, 127.0, 124.3, 85.6, 56.8, 50.8; HRMS (ESI, m/z) for $C_{22}H_{23}ClN_3$ calcd, 364.1580 [M+H]$^+$; found, 364.1568 [M+H]$^+$.

1,3-dibenzyl-2-(2,4-dichloro-3-pyridyl)-imidazolidine (**4n**). White solid, 222 mg, yield 56%. ^{1}H NMR (500 MHz, $CDCl_3$):δ (ppm) 8.30 (d, *J* = 8.0 Hz, 1H), 7.34 (d, *J* = 8.0 Hz, 1H), 7.28 (d, *J* = 9.5 Hz, 3H), 7.25-7.20 (m, 7H), 4.52 (s, 1H), 3.75 (d, *J* = 13.0 Hz, 2H), 3.46 (d, *J* = 13.0 Hz, 2H), 3.26-3.18 (m, 2H), 2.67-2.59 (m, 2H); ^{13}C NMR (126 MHz, $CDCl_3$):δ150.4, 149.8, 142.9, 138.4, 134.4, 128.3, 128.2, 127.1, 123.7, 82.9, 56.9, 50.9; HRMS (ESI, m/z) for $C_{22}H_{22}Cl_2N_3$ calcd, 398.1191 [M+H]$^+$; found, 398.1179 [M+H]$^+$.

1,3-dibenzyl-2-(4-bromo-3-pyridyl)-imidazolidine (**4o**). White solid, 265 mg, yield 65%. ^{1}H NMR (500 MHz, $CDCl_3$) :δ (ppm) 8.42 (d, *J* = 2.5 Hz, 1H), 7.86 (dd, *J* = 8.0, 2.5 Hz, 1H), 7.48 (d, *J* = 8.0 Hz, 1H), 7.26-7.18 (m, 10H), 3.89 (s, 1H), 3.71 (d, *J* = 13.0 Hz, 2H), 3.31 (d, *J* = 13.0 Hz, 2H), 3.22- 3.16 (m, 2H), 2.56 (q, *J* = 5.0 Hz, 2H); ^{13}C NMR (126 MHz, $CDCl_3$) :δ 151.2, 142.3, 139.6, 138.4, 136.2, 128.4, 128.2, 128.1, 127.0, 85.6, 56.8, 50.8; HRMS (ESI, m/z) for $C_{22}H_{23}BrN_3$ calcd, 408.1075 [M+H]$^+$; found, 408.0993 [M+H]$^+$.

1,3-dibenzyl-2-(3-bromo-5-pyridyl)-imidazolidine (**4p**). White solid, 313 mg, yield 77%. ^{1}H NMR (500 MHz, $CDCl_3$) :δ (ppm) 8.67-8.55 (m, 2H), 8.11 (s, 1H), 7.3-7.27 (m, 4H), 7.23 (d, *J* = 7.5 Hz, 6H), 3.93 (s, 1H), 3.76 (d, *J* = 13.0 Hz, 2H), 3.37 (d, *J* = 13.0 Hz, 2H), 3.24 (d, *J* = 4.0 Hz, 2H), 2.61 (d, *J* = 4.0 Hz, 2H); ^{13}C NMR (126 MHz, $CDCl_3$): δ 151.0, 149.0, 139.4, 138.7, 138.3, 128.5, 128.2, 127.1, 120.8, 85.8, 57.0, 50.9; HRMS (ESI, m/z) for $C_{22}H_{23}BrN_3$ calcd, 408.1075 [M+H]$^+$; found, 408.0991 [M+H]$^+$.

1,3-dibenzyl-2-(3-bromo-2-pyridyl)-imidazolidine (**4q**). White solid, 171 mg, yield 42%. ^{1}H NMR (500 MHz, $CDCl_3$): δ (ppm) 7.96 (d, *J* = 7.5 Hz, 1H), 7.62 (t, *J* = 7.5 Hz, 1H), 7.42 (d, *J* = 7.5 Hz, 1H), 7.29-7.26 (m, 7H), 7.26-7.19 (m, 3H), 4.15 (s, 1H), 3.86 (d, *J* = 14.0 Hz, 2H), 3.46 (d, *J* = 14.0 Hz, 2H), 3.24 (q, *J* = 5.0 Hz, 2H), 2.61 (q, *J* = 5.0 Hz, 2H); ^{13}C NMR (126 MHz, $CDCl_3$): δ 163.7, 140.2, 139.1, 138.8, 128.4, 128.1, 127.3, 126.9, 121.9, 88.4, 57.1, 51.0; HRMS (ESI, m/z) for $C_{22}H_{23}BrN_3$ calcd, 408.1075 [M+H]$^+$; found, 408.0996 [M+H]$^+$.

1,3-dibenzyl-2-pyrimidine-imidazolidine (**4r**). White solid, 23 mg, yield 7%. ^{1}H NMR (500 MHz, $CDCl_3$) :δ (ppm) 9.12 (s, 1H), 8.82 (s, 2H), 7.26-7.19 (m, 10H), 3.95 (s, 1H), 3.72 (d, *J* = 13.0 Hz, 2H), 3.41 (d, *J* = 13.0 Hz, 2H), 3.24 (d, *J* = 4.0 Hz, 2H), 2.62 (d, *J* = 4.0 Hz, 2H); ^{13}C NMR (126 MHz, $CDCl_3$): δ 158.7, 158.0, 138.1, 134.6, 128.4, 128.3, 127.2, 84.6, 57.1, 51.1; HRMS (ESI, m/z) for $C_{21}H_{23}N_4$ calcd, 331.1923 [M+H]$^+$; found, 331.1914 [M+H]$^+$.

1,3-diphenyl-2-furan-imidazolidine (**6a,** CAS No. 94378-00-4)[3]. White solid, 116 mg, yield 40%. ^{1}H NMR (500 MHz, $CDCl_3$) :δ (ppm) 7.25-7.20 (m, 5H), 6.76 (d, *J* = 1.0 Hz, 6H), 6.32 (d, *J* = 3.5 Hz, 1H), 6.21 (s, 1H), 6.15 (s, 1H), 3.80 (s, 4H); ^{13}C NMR (126 MHz, $CDCl_3$):δ 153.2, 145.2, 142.1, 129.1, 117.8, 113.2, 110.0, 108.8, 70.4, 45.6.

1,3-diphenyl-2-(2-chloro-5-thiazolyl)-imidazolidine (**6b**). White solid, 160 mg, yield 47%. ^{1}H NMR (500 MHz, $CDCl_3$) :δ (ppm) 7.58 (s, 1H), 7.28 (s, 1H), 7.21-7.17 (m, 1H), 6.84 (s, 2H), 6.73 (d, *J* = 8.0 Hz, 4H), 6.67 (d, *J* = 8.0 Hz, 1H), 6.32 (s, 1H), 3.71 (s, 4H), 3.41 (s, 1H); ^{13}C NMR (126 MHz, $CDCl_3$) : δ 151.9, 144.5, 141.3, 139.5, 129.4, 119.0, 113.9, 71.2, 45.4. HRMS (ESI, m/z) for $C_{18}H_{17}ClN_3S$ calcd, 342.0832 $[M+H]^+$; found, 342.0822 $[M+H]^+$.

1,3-diphenyl-2-(5-chloro-2-fluoro-phenyl)-imidazolidine (**6c**). White solid, 169 mg, yield 48%. ^{1}H NMR (500 MHz, $CDCl_3$): δ (ppm) 7.29 (dd, *J* = 6.0, 3.0 Hz, 1H), 7.26 (s, 2H), 7.22 (t, *J* = 8.0 Hz, 3H), 7.15-7.05 (m, 1H), 7.01-6.91 (m, 1H), 6.76 (t, *J* = 7.5 Hz, 2H), 6.71 (d, *J* = 8.0 Hz, 3H), 6.29 (s, 1H), 4.04-4.01 (m, 2H), 3.80-3.77 (m, 2H); ^{13}C NMR (126 MHz, $CDCl_3$): δ 159.5 (d, *J* = 260 Hz), 144.9, 130.7 (d, *J* =13.9 Hz), 129.8 (d, *J* = 260 Hz), 129.3, 128.9 (d, *J* = 8.0 Hz), 118.1, 116.8 (d, *J* = 25.2 Hz), 113.1 (d, *J* = 1.26 Hz), 70.3, 46.4. HRMS (ESI, m/z) for $C_{21}H_{19}ClFN_2$ calcd, 353.1221 $[M+H]^+$; found, 353.1205 $[M+H]^+$.

1,3-diphenyl-2-(2,6-dichloro-3-pyridyl)-imidazolidine (**6d**). White solid, 188 mg, yield 51%. ^{1}H NMR (500 MHz, $CDCl_3$): δ (ppm) 7.66 (d, *J* = 8.0 Hz, 1H), 7.26-7.12 (m, 6H), 6.83-6.74 (m, 5H), 6.25 (s, 1H), 3.99-3.96 (m, 2H), 3.76-3.73 (m, 2H); ^{13}C NMR (126 MHz, $CDCl_3$) :149.7, 149.3, 145.2, 140.5, 135.1, 129.4, 124.0, 119.1, 114.2, 73.8, 47.3. HRMS (ESI, m/z) for $C_{20}H_{18}Cl_2N_3$ calcd, 370.0878 $[M+H]^+$; found, 370.0867 $[M+H]^+$.

1,3-diethyl-2-(2-chloro-4-pyridyl)-imidazolidine (**7a**). Yellow oil, 90 mg, yield 38%. ^{1}H NMR (500 MHz, $CDCl_3$): δ (ppm) 8.31-8.13 (m, 1H), 7.37 (s, 1H), 7.26 (dd, *J* = 5.0, 1.3 Hz, 1H), 3.49 (s, 1H), 3.30 (q, *J* = 5.0 Hz, 2H), 2.51-2.46 (m, 2H), 2.43-2.36 (m, 2H), 2.20-2.13 (m, 2H), 0.89 (t, *J* = 7.0 Hz, 6H); ^{13}C NMR (126 MHz, $CDCl_3$): δ 153.8, 150.4, 148.4, 123.4, 121.9, 86.4, 49.3, 45.9, 12.6. HRMS (ESI, m/z) for $C_{12}H_{19}ClN_3$ calcd, 240.1267 $[M+H]^+$; found, 240.1260 $[M+H]^+$.

1,3-diethyl-2- (5-chloro-2-fluoro-phenyl)-imidazolidine (**7b**). Yellow oil, 110 mg, yield 43%. ^{1}H NMR (500 MHz, $CDCl_3$): δ (ppm) 7.65 (dd, *J* = 6.0, 3.0 Hz, 1H), 7.12-7.08 (m, 1H), 6.86-6.82 (m, 1H), 3.99 (s, 1H), 3.30-3.27 (m, 2H), 2.48-2.42 (m, 4H), 2.20-2.16 (m, 2H), 0.90 (t, *J* = 7.0 Hz, 6H); ^{13}C NMR (126 MHz, $CDCl_3$) :δ 160.7 (d, *J* = 246 Hz), 130.2 (d, *J* =7.2 Hz), 130.1 (d, *J* = 4.5 Hz), 129.6 d, *J* = 3.0 Hz, 129.2 (d, *J* = 8.7 Hz), 116.2 ((d, *J* = 24.4 Hz), 80.1, 50.2, 46.8, 13.5. HRMS (ESI, m/z) for $C_{13}H_{19}ClFN_2$ calcd, 257.1221 $[M+H]^+$; found, 257.1213 $[M+H]^+$.

1,3-dimethyl-2- (5-chloro-2-fluoro-phenyl)-imidazolidine (**8a**). Yellow oil, 100 mg, yield 44%. ^{1}H NMR (500 MHz, $CDCl_3$) :δ (ppm) 7.70 (dd, *J* = 6.0, 3.0 Hz, 1H), 7.24-7.20 (m, 1H), 6.98-6.95 (m, 1H), 3.85 (s, 1H), 3.39-3.35 (m, 2H), 2.62 (qd, *J* = 6.0, 2.0 Hz, 2H), 2.24 (d, *J* = 1.0 Hz, 6H); ^{13}C NMR (126 MHz, $CDCl_3$) :δ 160.7 (d, *J* = 246 Hz), 129.9 (d, *J* = 3.2 Hz), 129.7 (d, *J* = 4.4 Hz), 129.5 (d, *J* = 8.7 Hz), 128.8 (d, *J* = 12.5 Hz), 116.4 (d, *J* = 24.6 Hz) , 82.8, 53.3, 39.4. HRMS (ESI, m/z) for $C_{11}H_{15}ClFN_2$ calcd, 229.0908 $[M+H]^+$; found, 229.0889 $[M+H]^+$.

2. NMR spectra for reported compounds

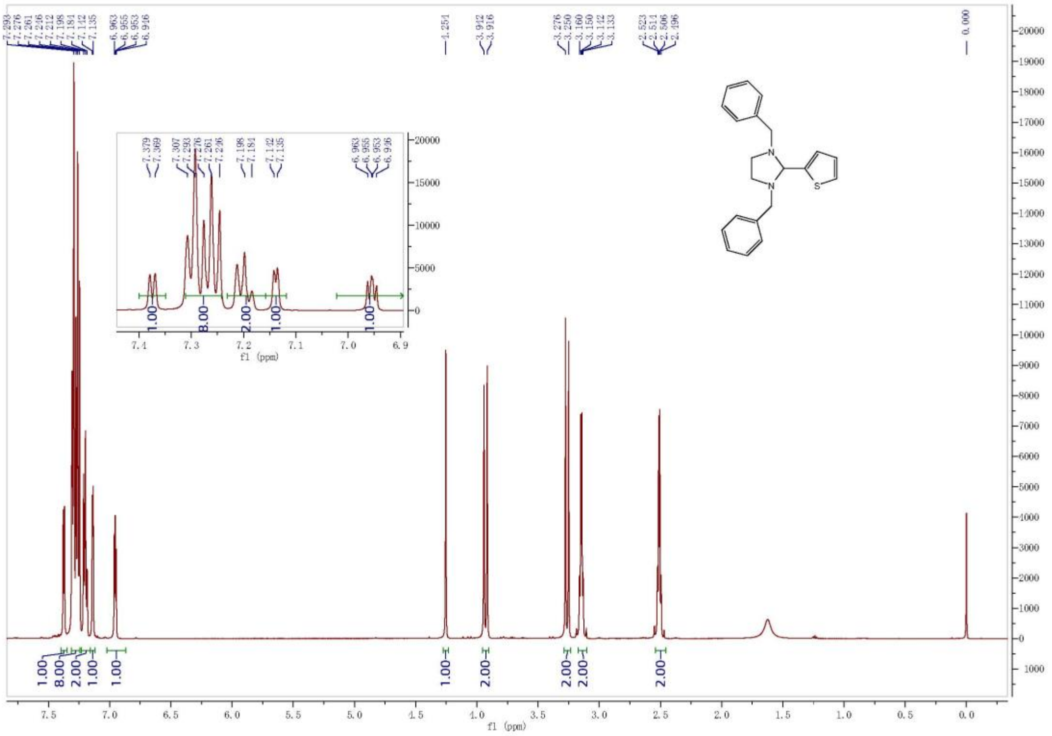

Figure 1 ^{1}H-NMR spectrum of compound **4c**

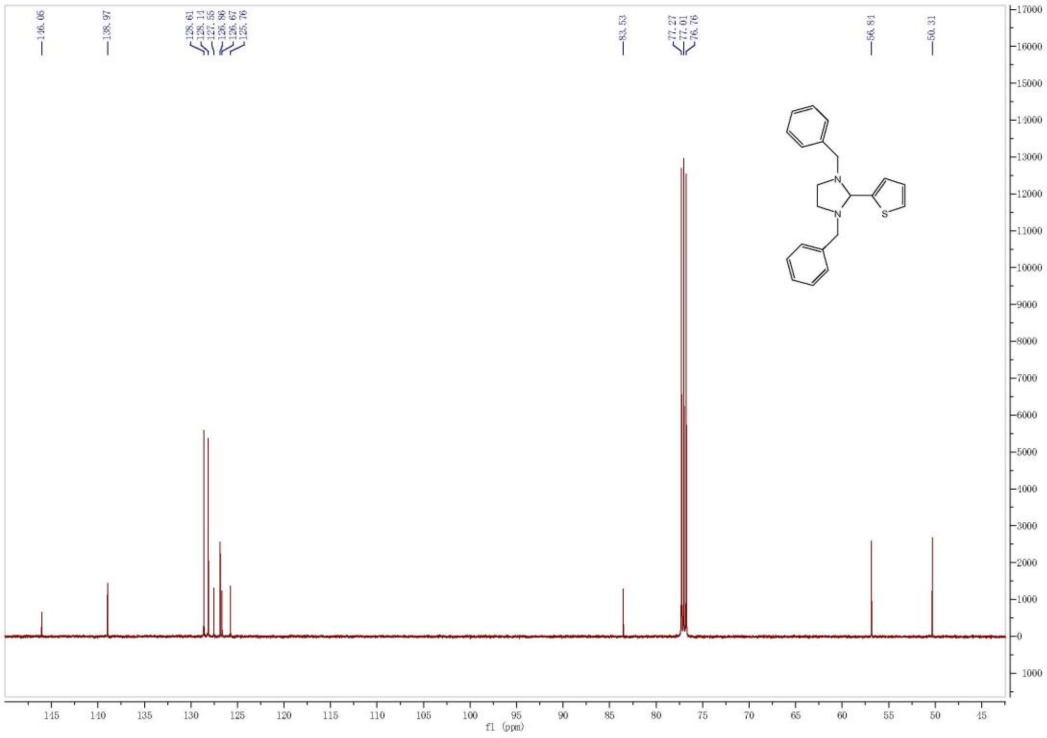

Figure 2 ^{13}C-NMR spectrum of compound **4c**

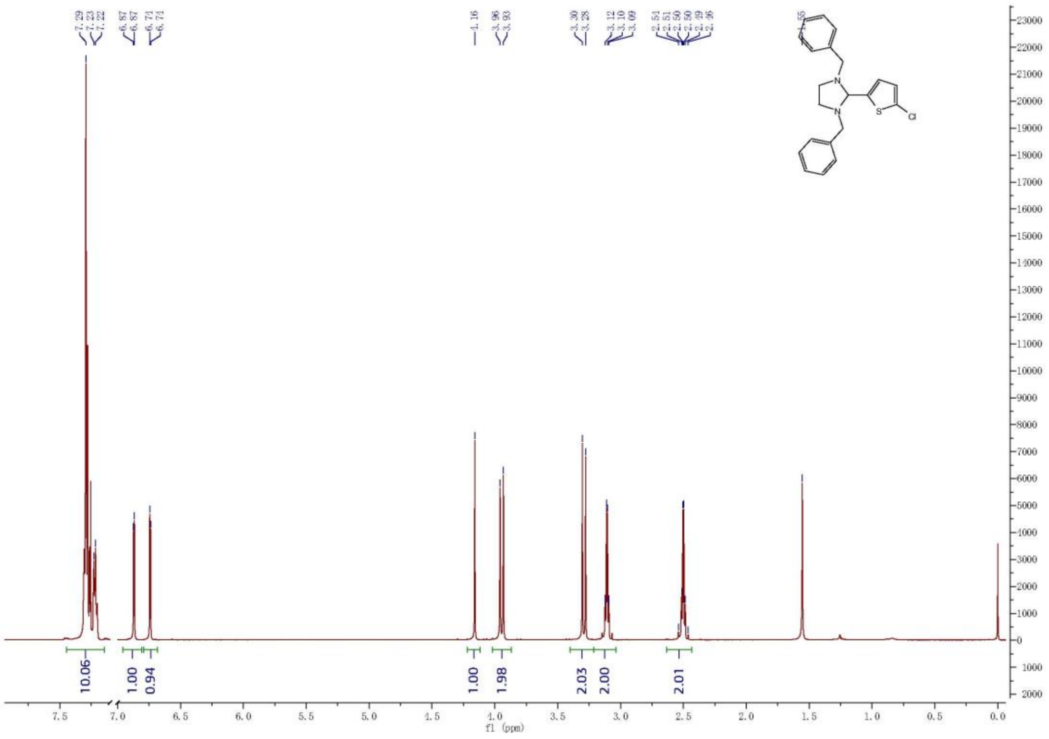

Figure 3 ^{1}H-NMR spectrum of compound **4d**

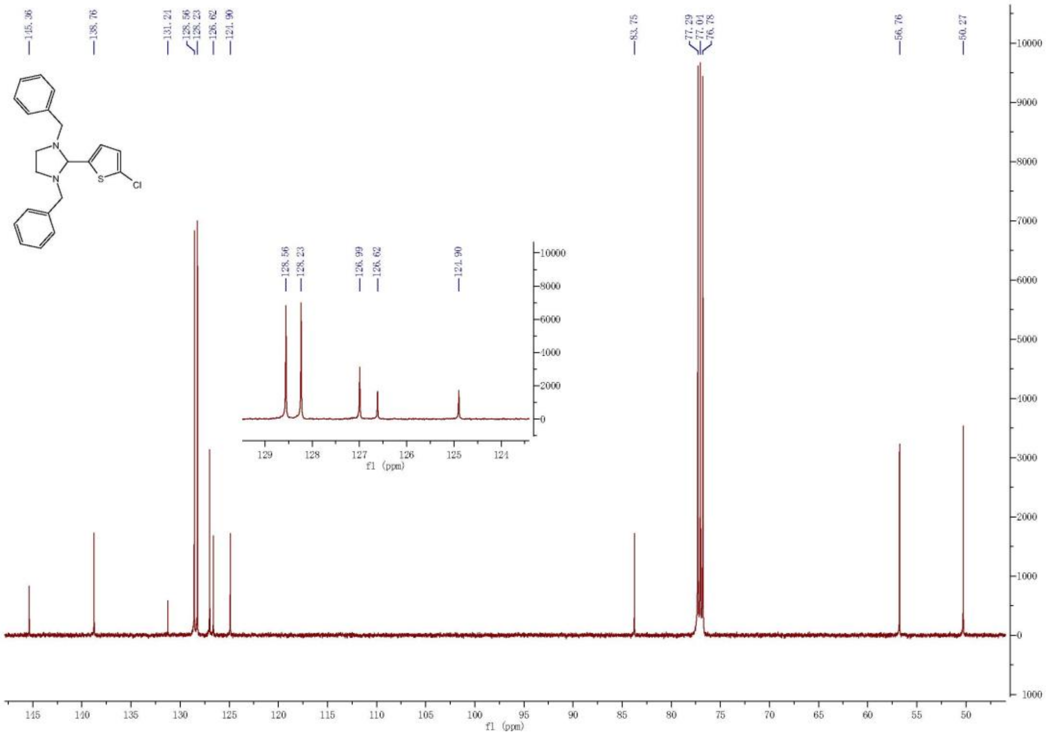

Figure 4 ^{13}C-NMR spectrum of compound **4d**

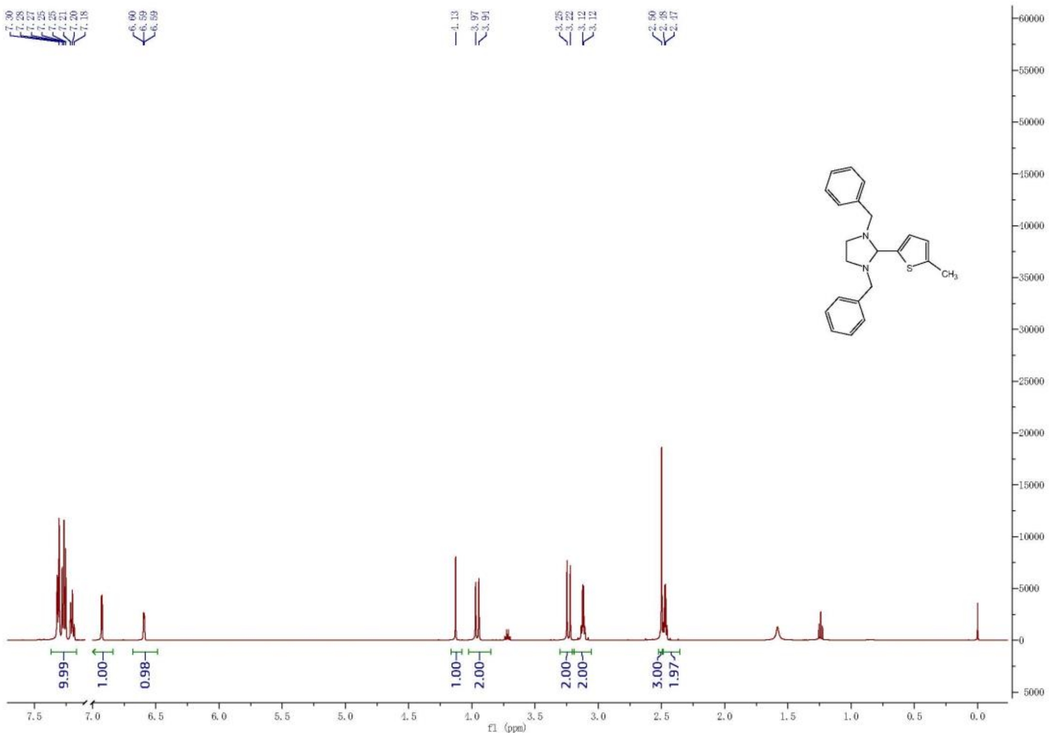

Figure 5 ^{1}H-NMR spectrum of compound **4e**

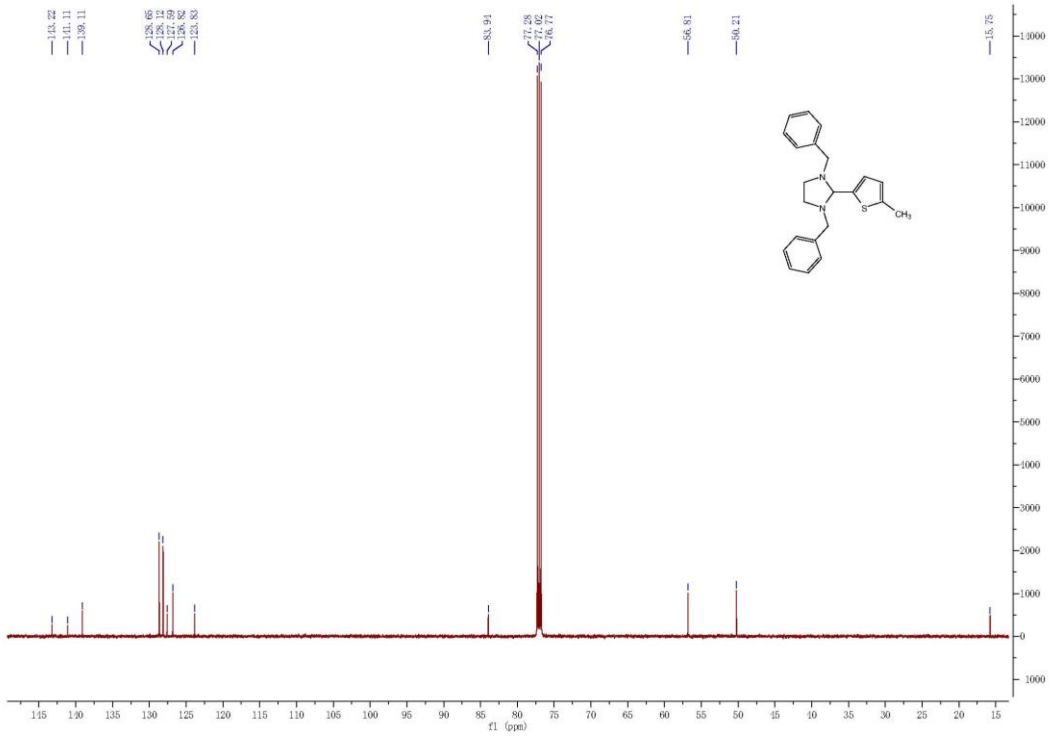

Figure 6 ^{13}C-NMR spectrum of compound **4e**

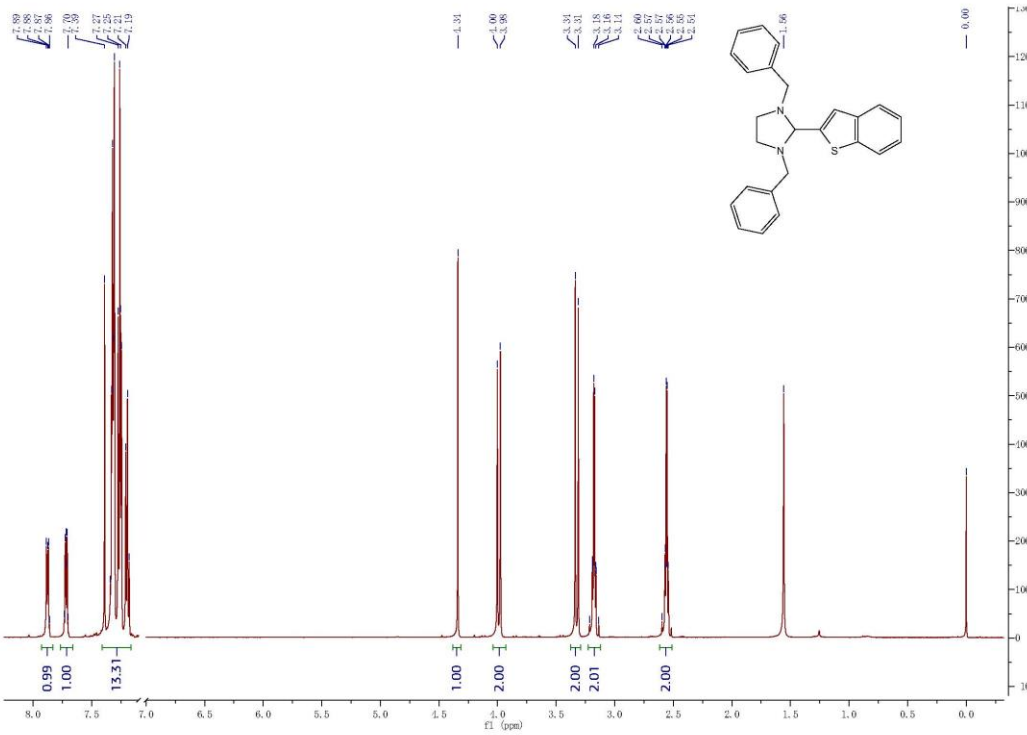

Figure 7 ^{1}H-NMR spectrum of compound **4f**

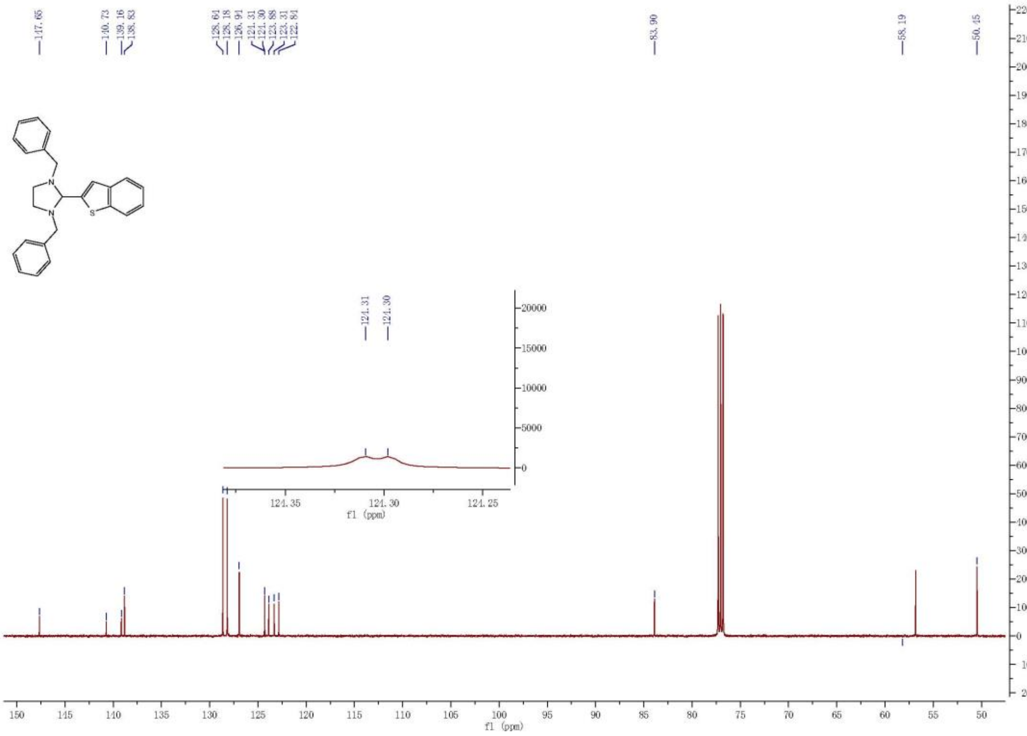

Figure 8 ^{13}C-NMR spectrum of compound **4f**

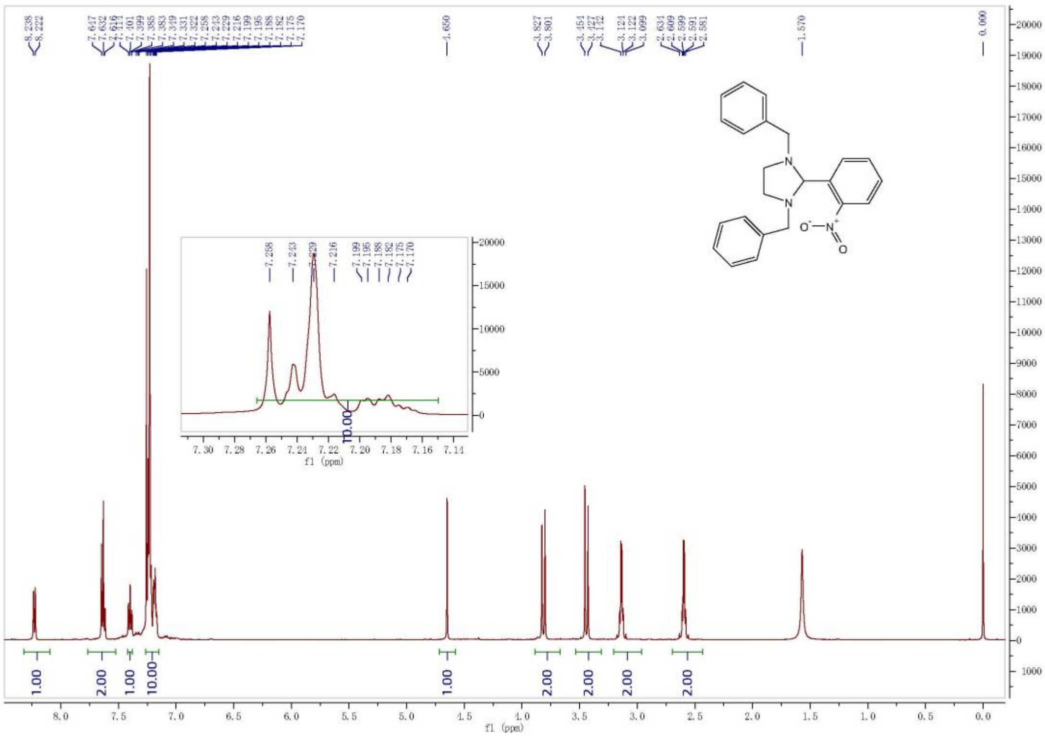

Figure 9 ^{1}H-NMR spectrum of compound **4g**

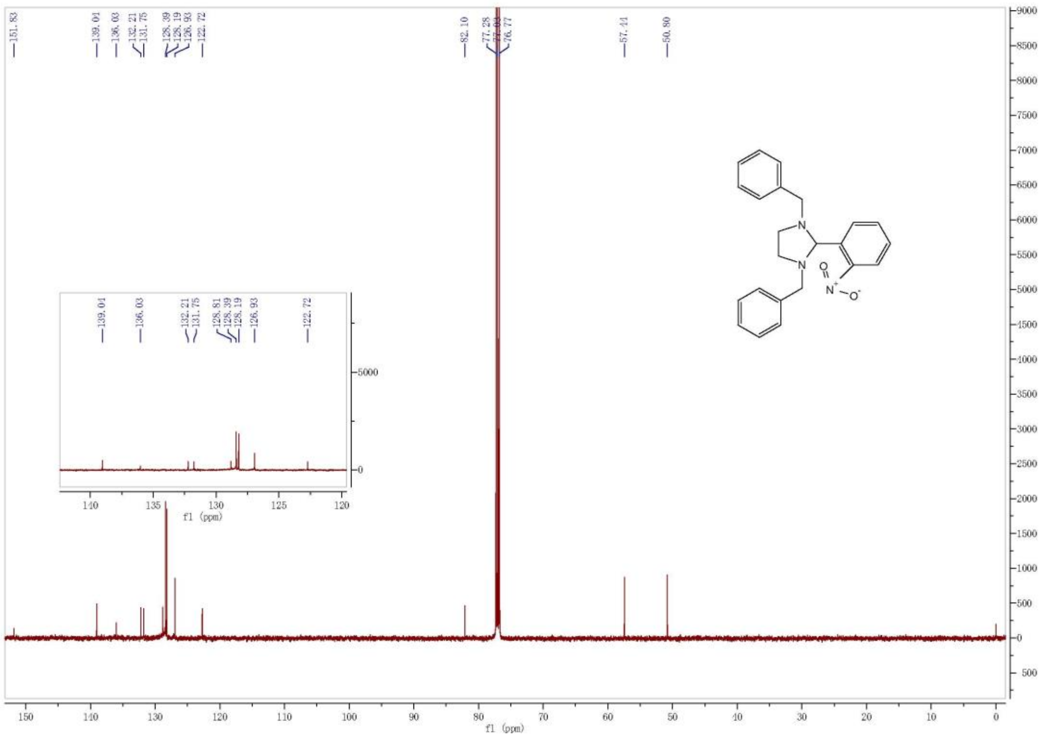

Figure 10 ^{13}C-NMR spectrum of compound **4g**

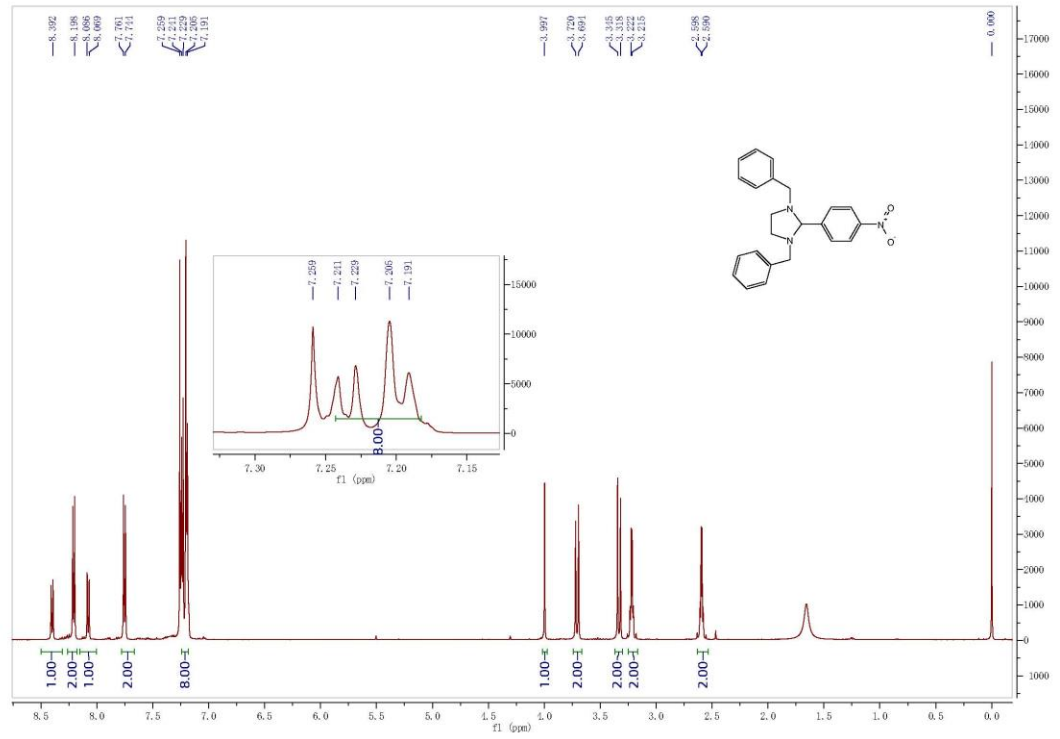

Figure 11 ^{1}H-NMR spectrum of compound **4h**

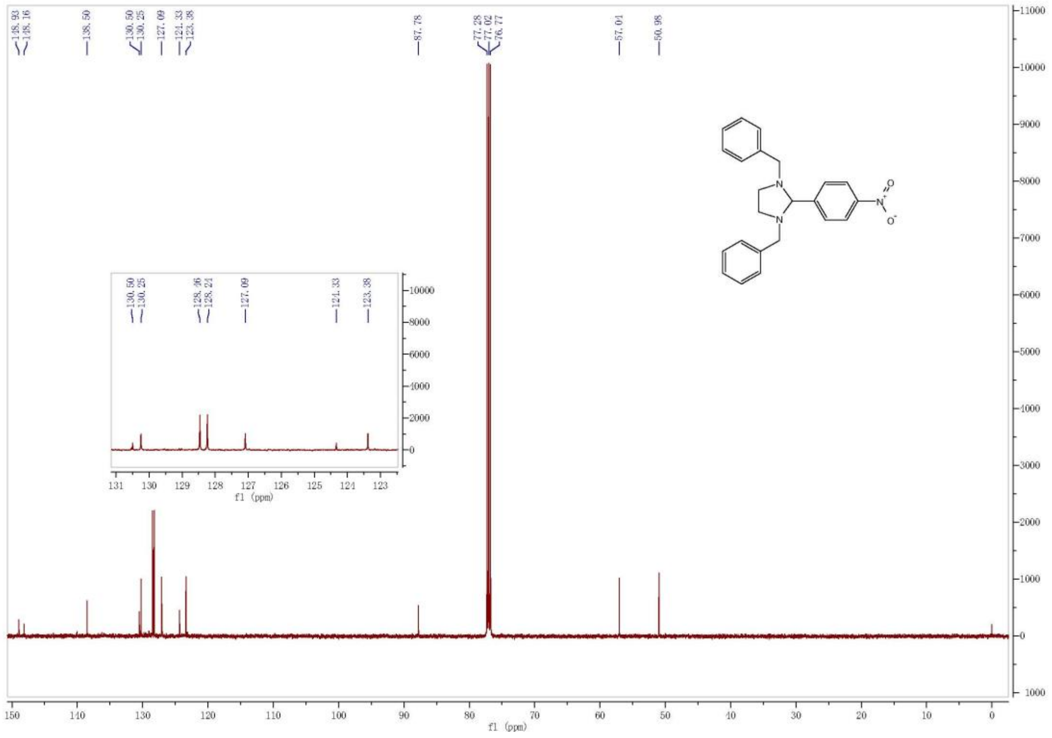

Figure 12 ^{13}C-NMR spectrum of compound **4h**

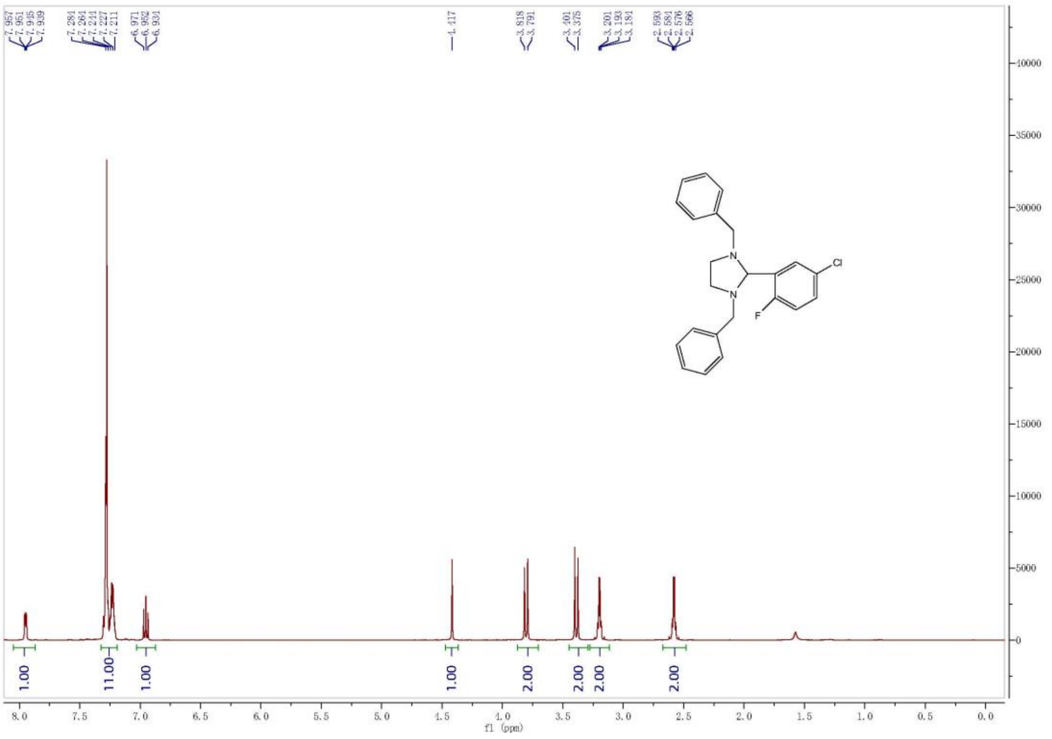

Figure 13 ^{1}H-NMR spectrum of compound **4i**

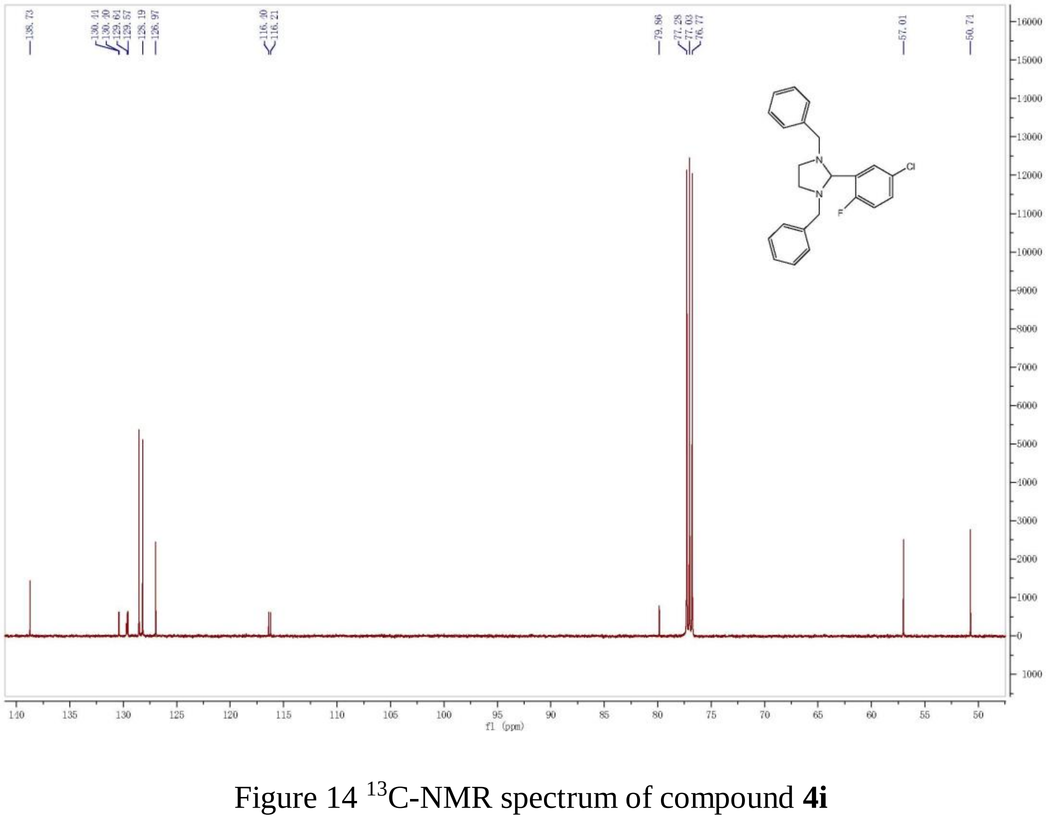

Figure 14 ^{13}C-NMR spectrum of compound **4i**

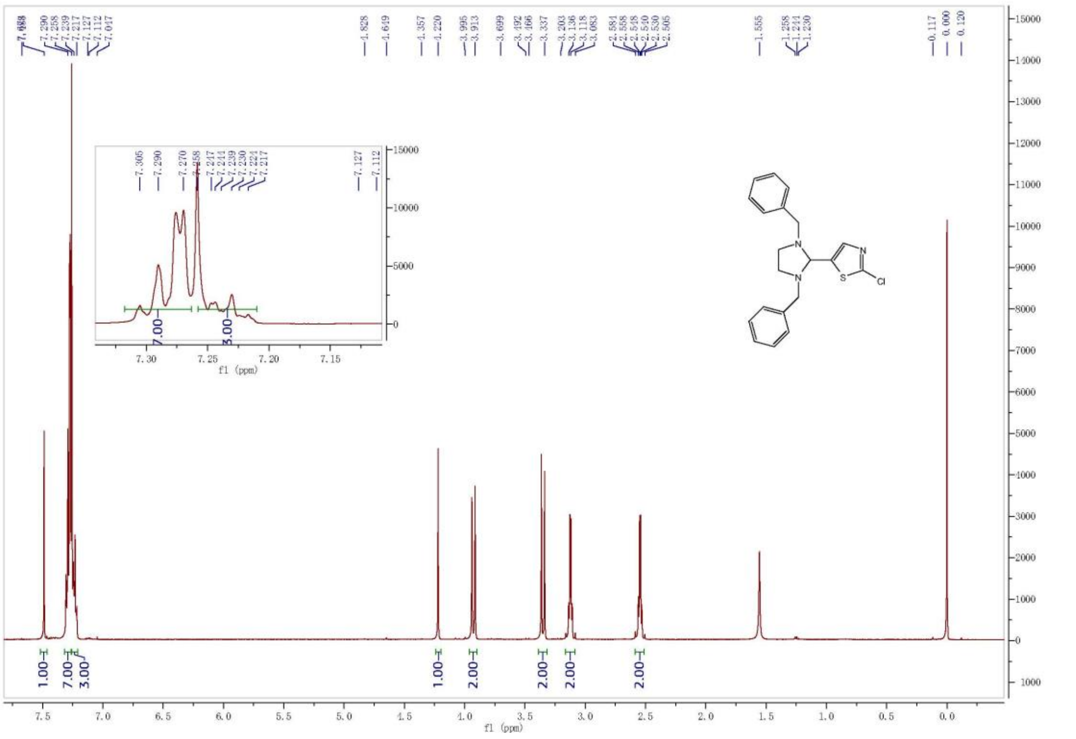

Figure 15 ^{1}H-NMR spectrum of compound **4j**

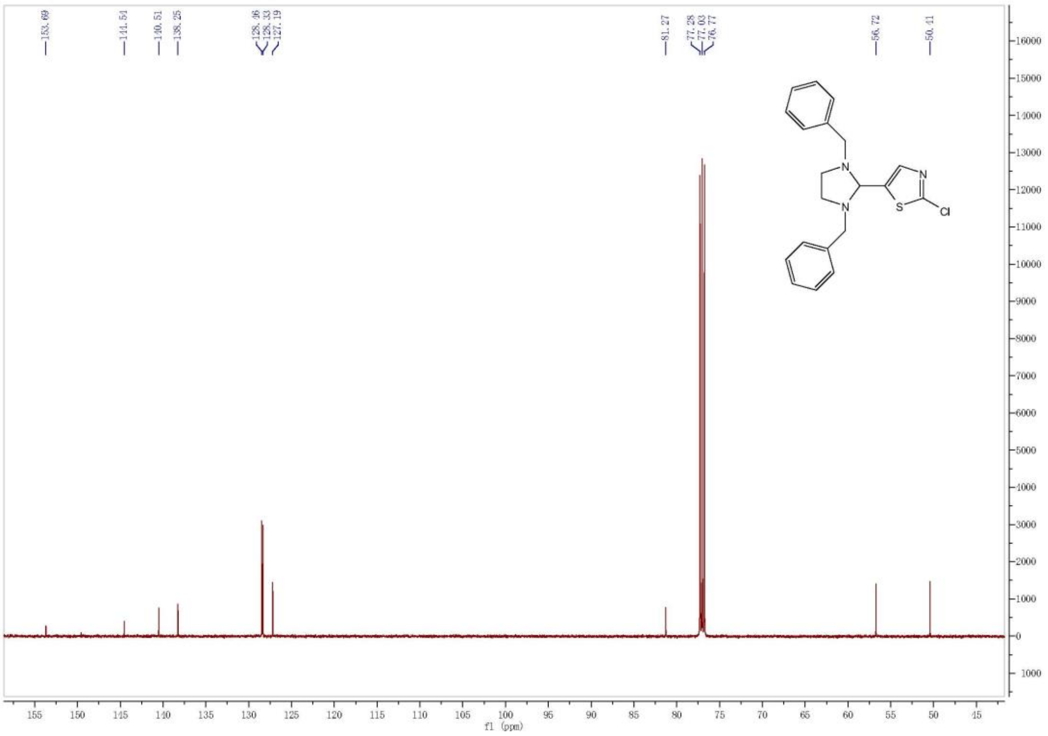

Figure 16 ^{13}C-NMR spectrum of compound **4j**

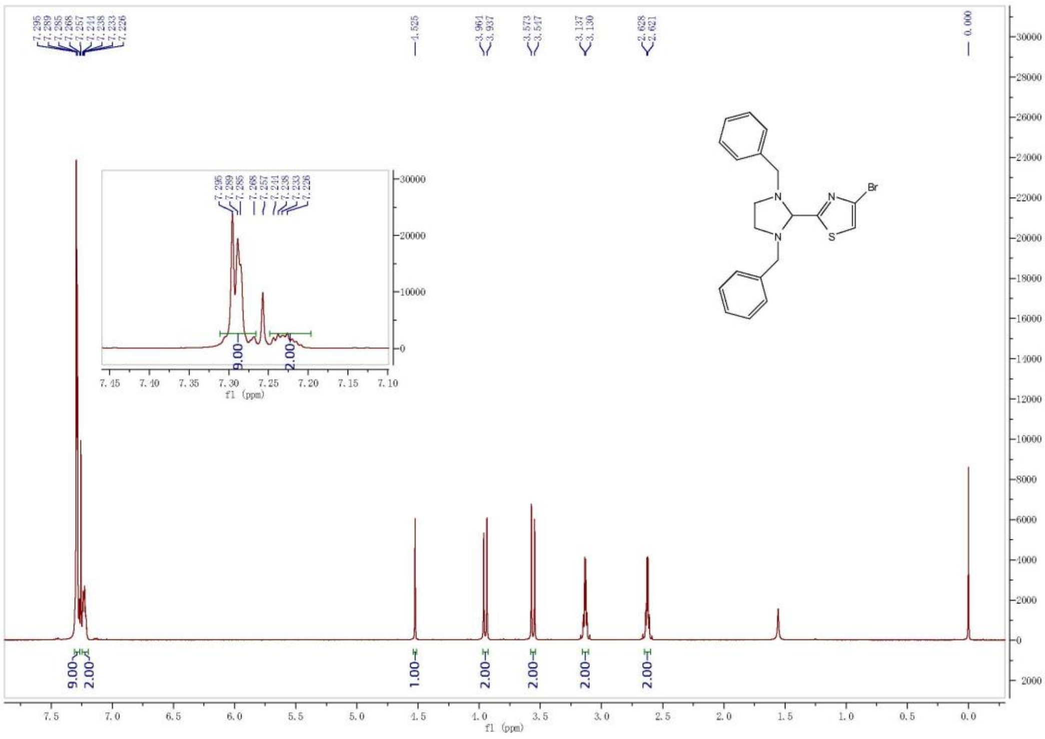

Figure 17 ^{1}H-NMR spectrum of compound **4k**

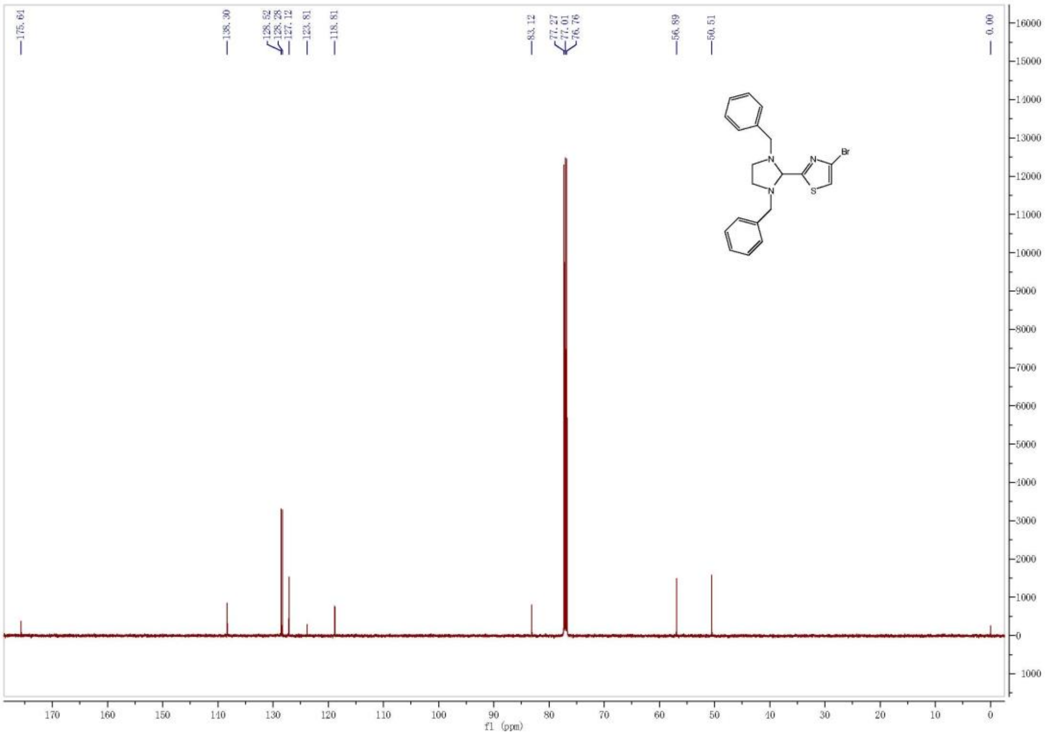

Figure 18 ^{13}C-NMR spectrum of compound **4k**

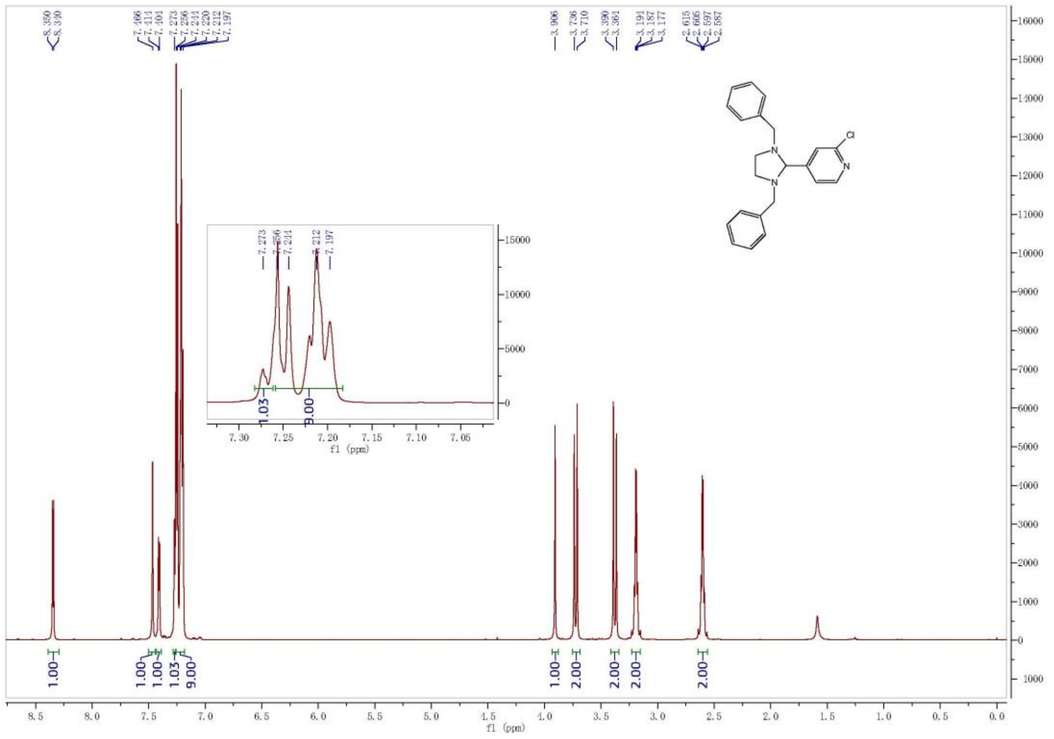

Figure19 ^{1}H-NMR spectrum of compound **4l**

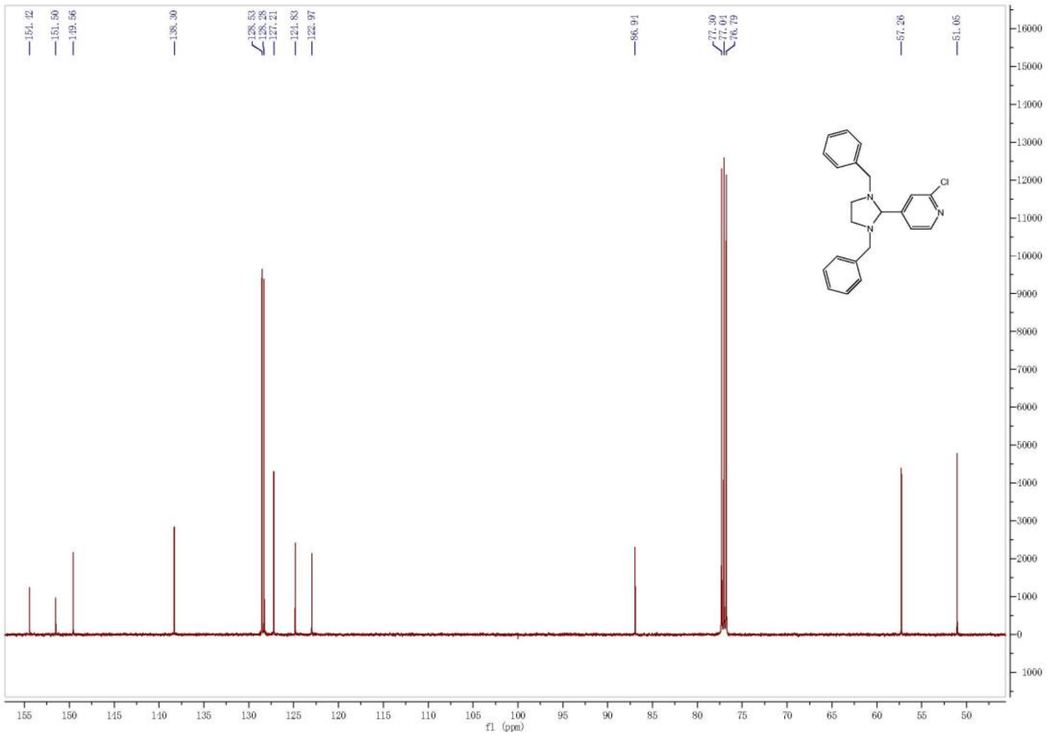

Figure 20 ^{13}C-NMR spectrum of compound **4l**

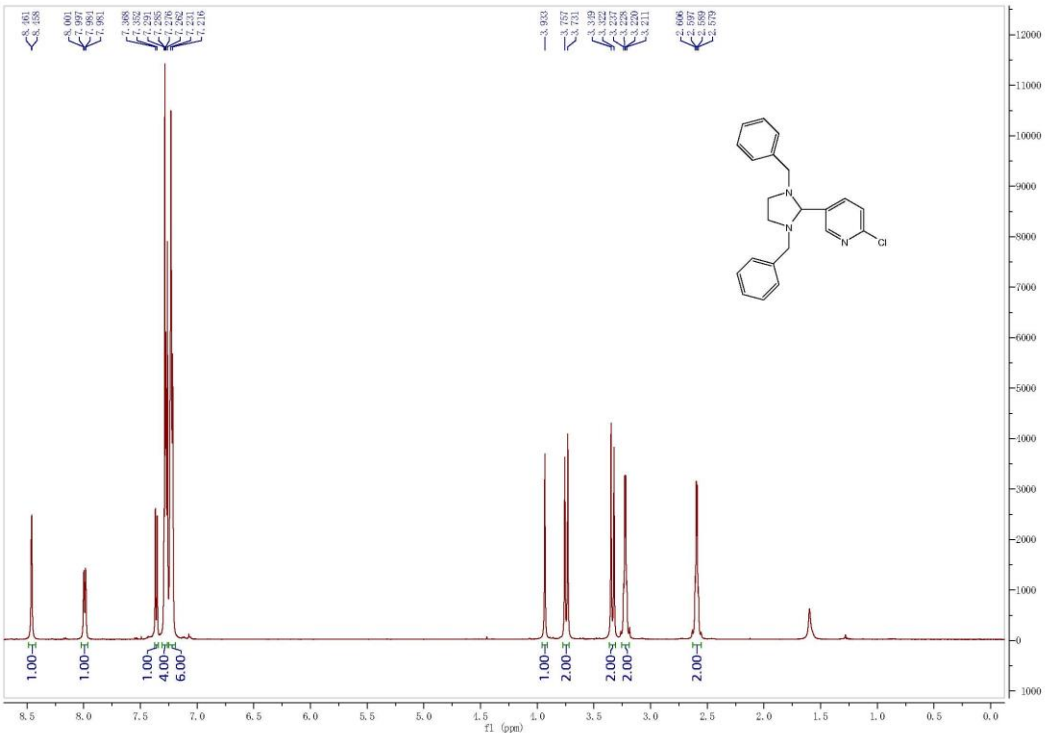

Figure 21 ^{1}H-NMR spectrum of compound **4m**

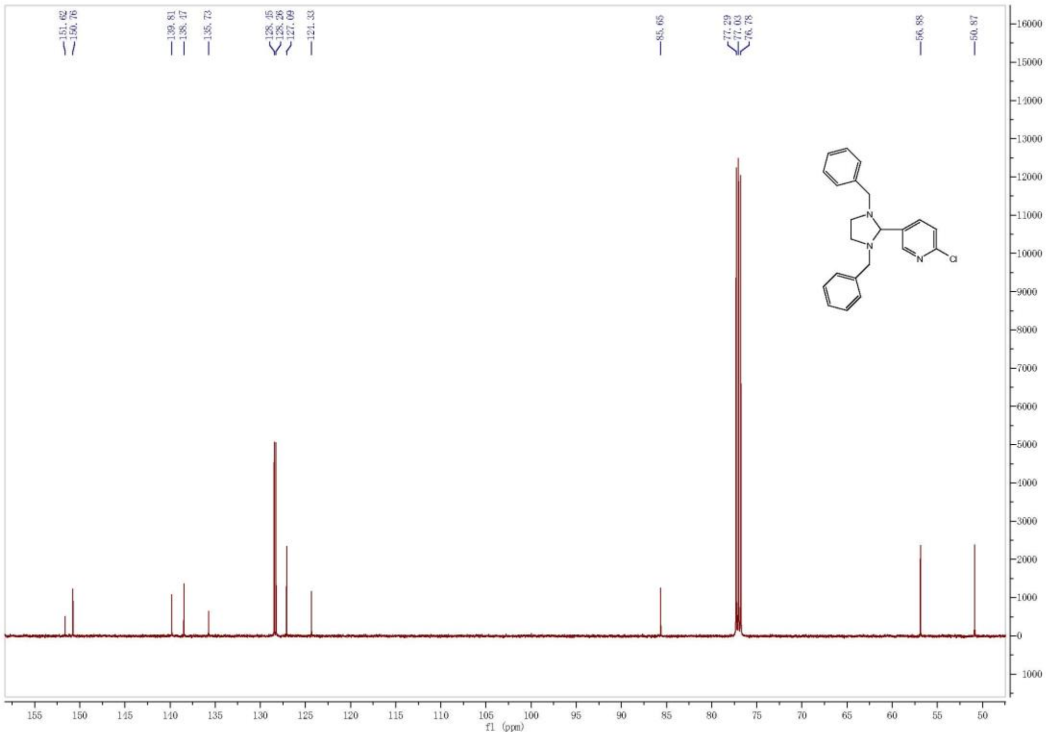

Figure 22 ^{13}C-NMR spectrum of compound **4m**

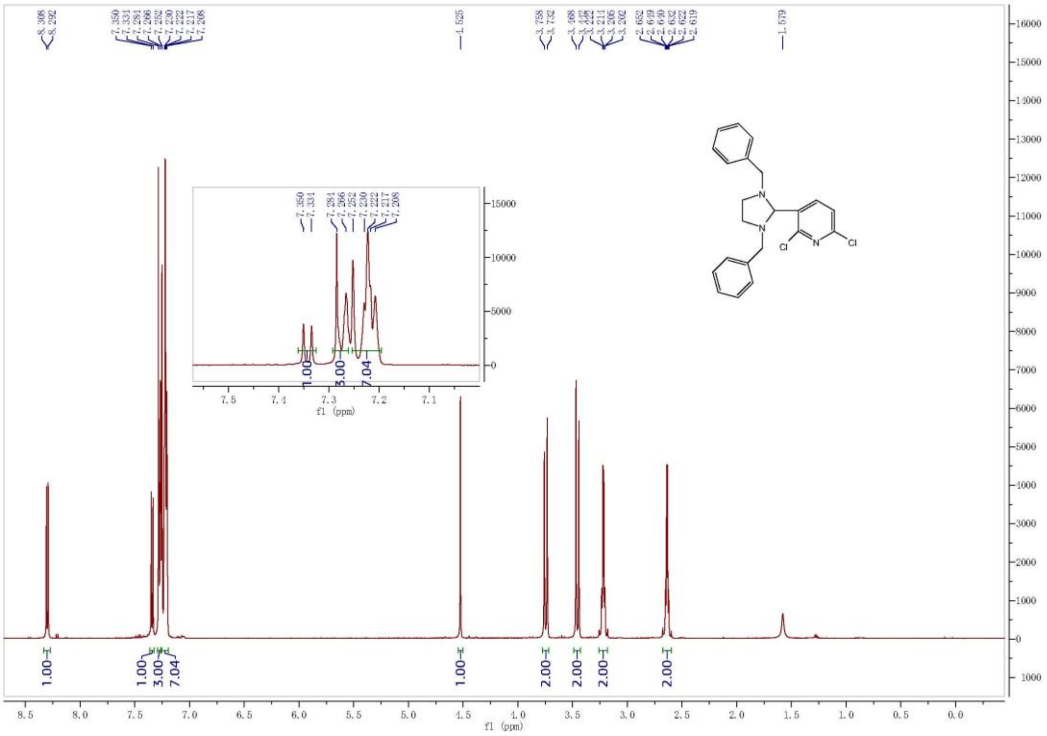

Figure 23 ^{1}H-NMR spectrum of compound **4n**

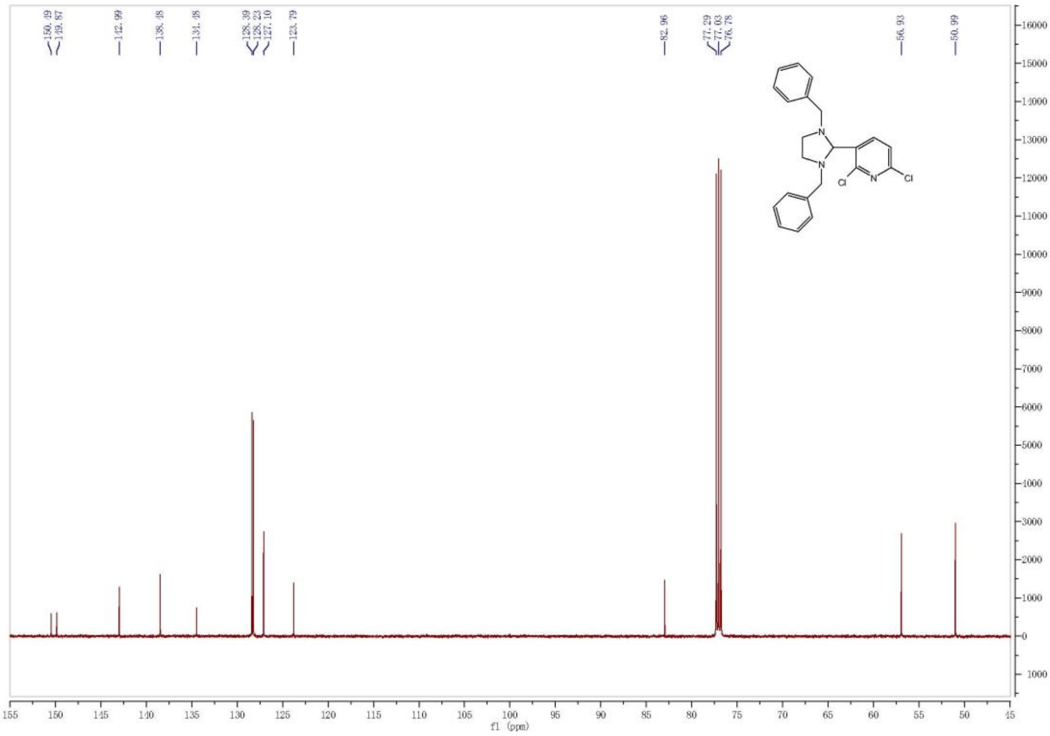

Figure 24 ^{13}C-NMR spectrum of compound **4n**

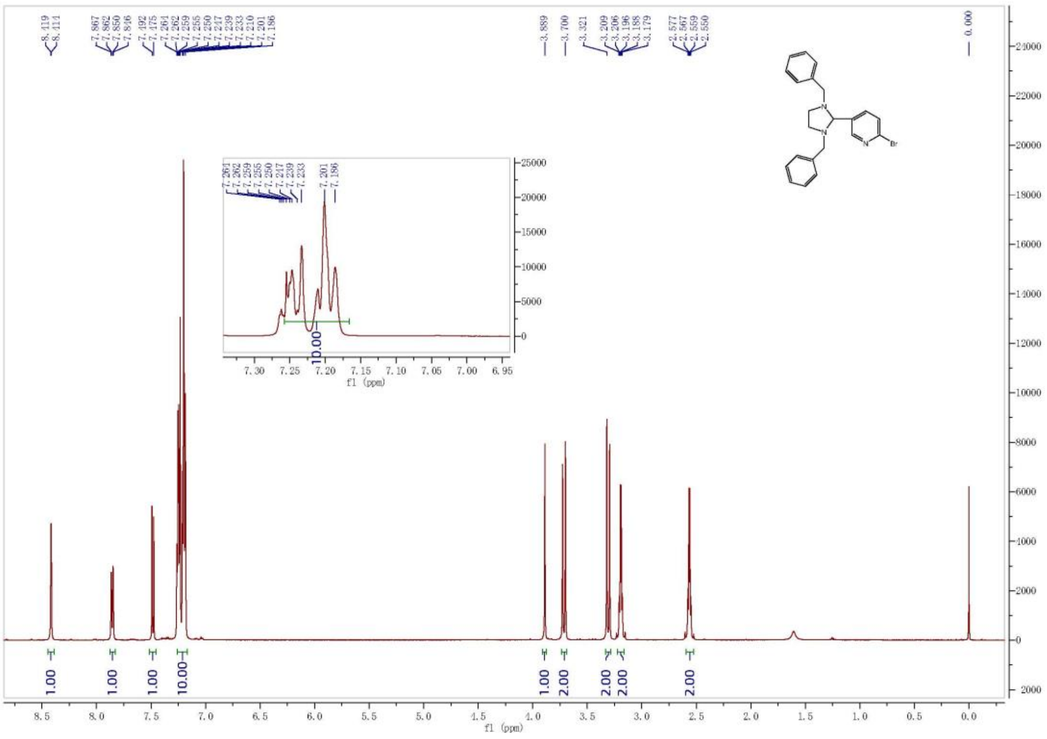

Figure 25 ^{1}H-NMR spectrum of compound **4o**

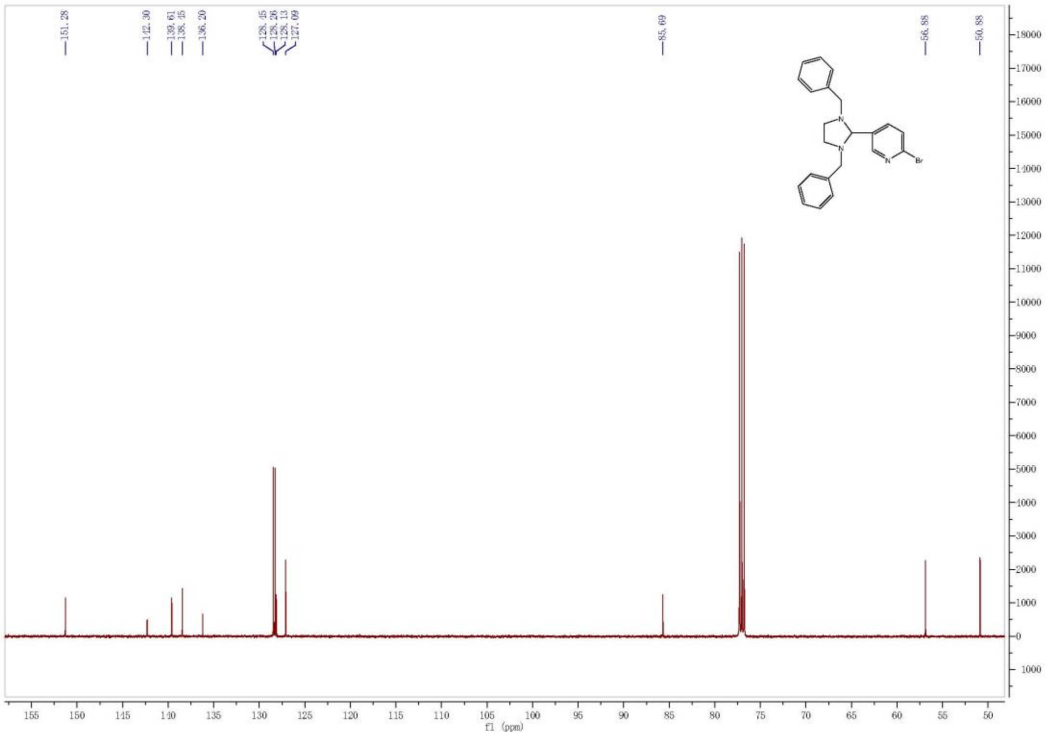

Figure 26 ^{13}C-NMR spectrum of compound **4o**

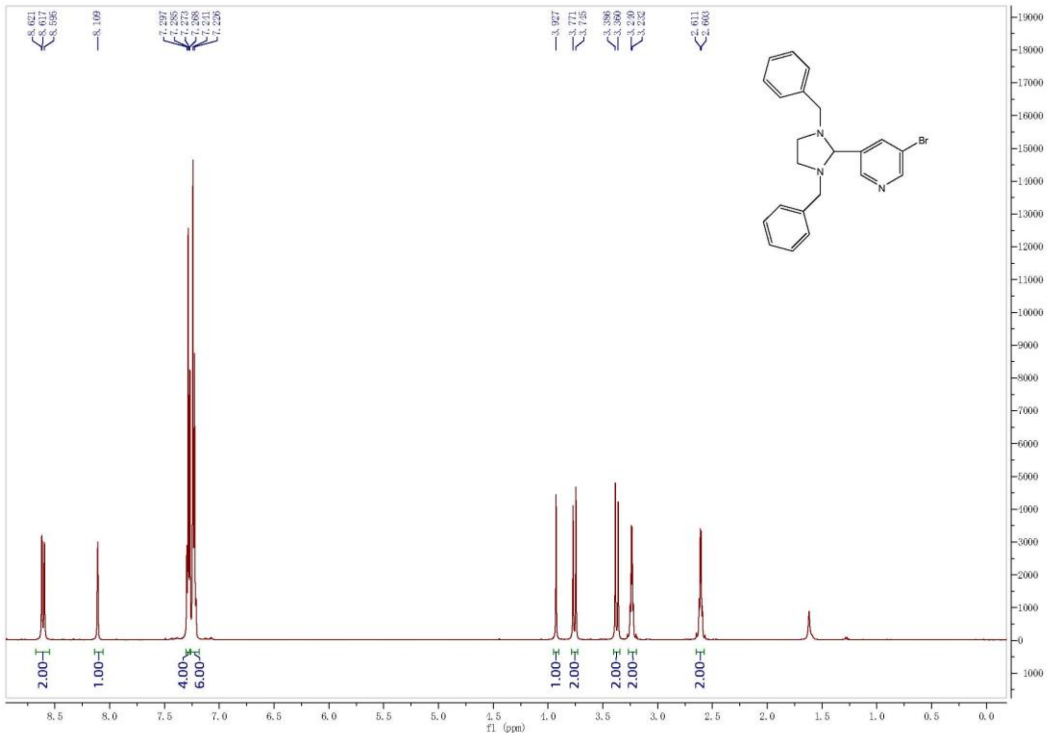

Figure 27 ^{1}H-NMR spectrum of compound **4p**

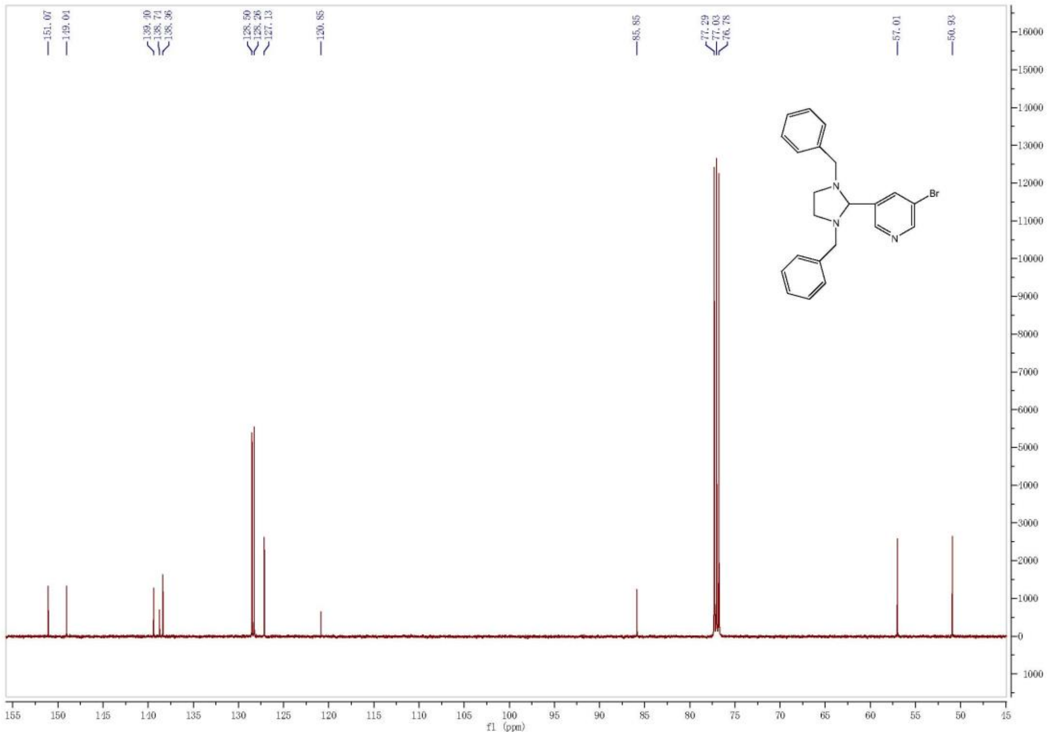

Figure 28 ^{13}C-NMR spectrum of compound **4p**

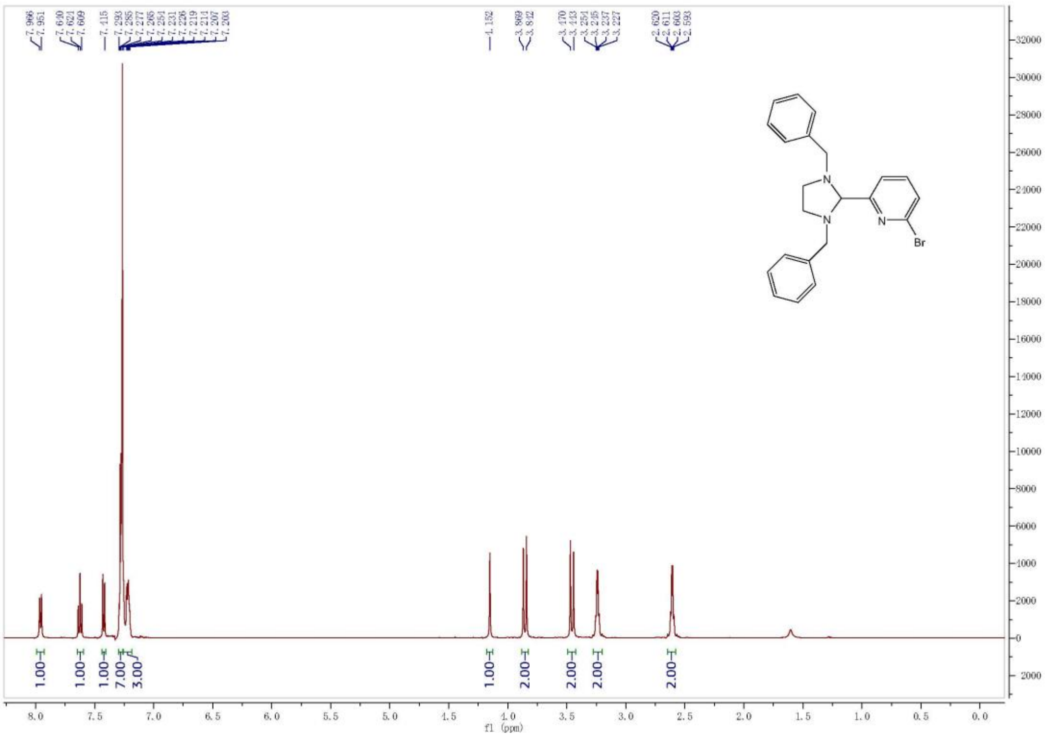

Figure 29 ^{1}H-NMR spectrum of compound **4q**

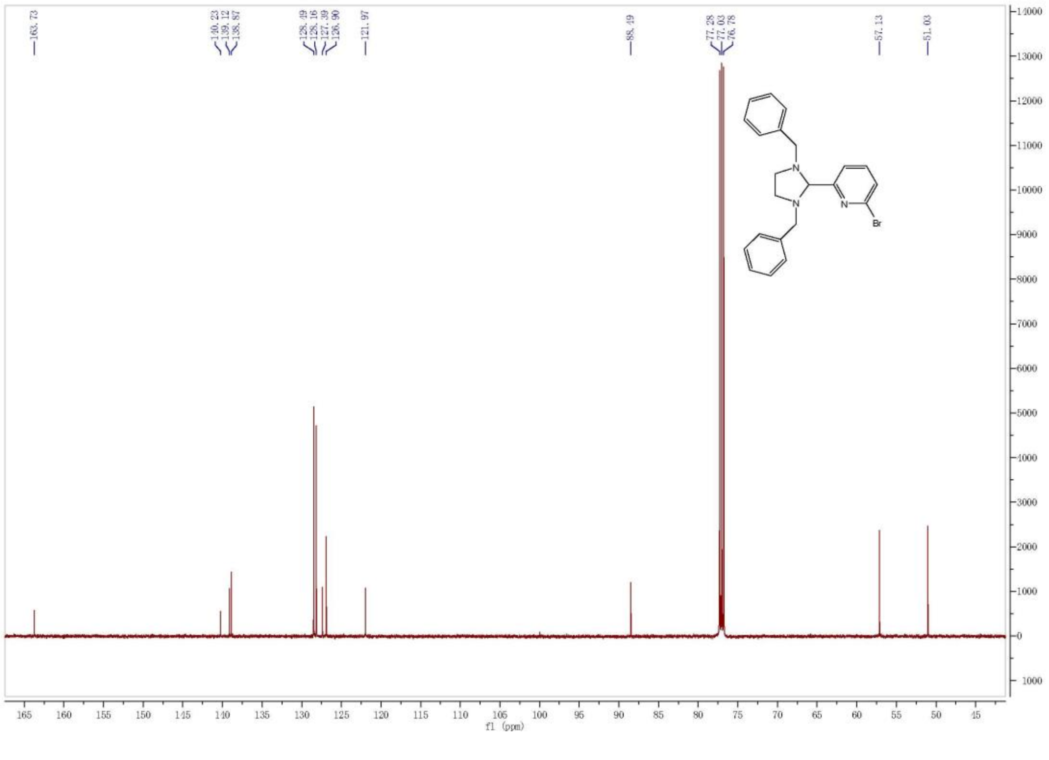

Figure 30 ^{13}C-NMR spectrum of compound **4q**

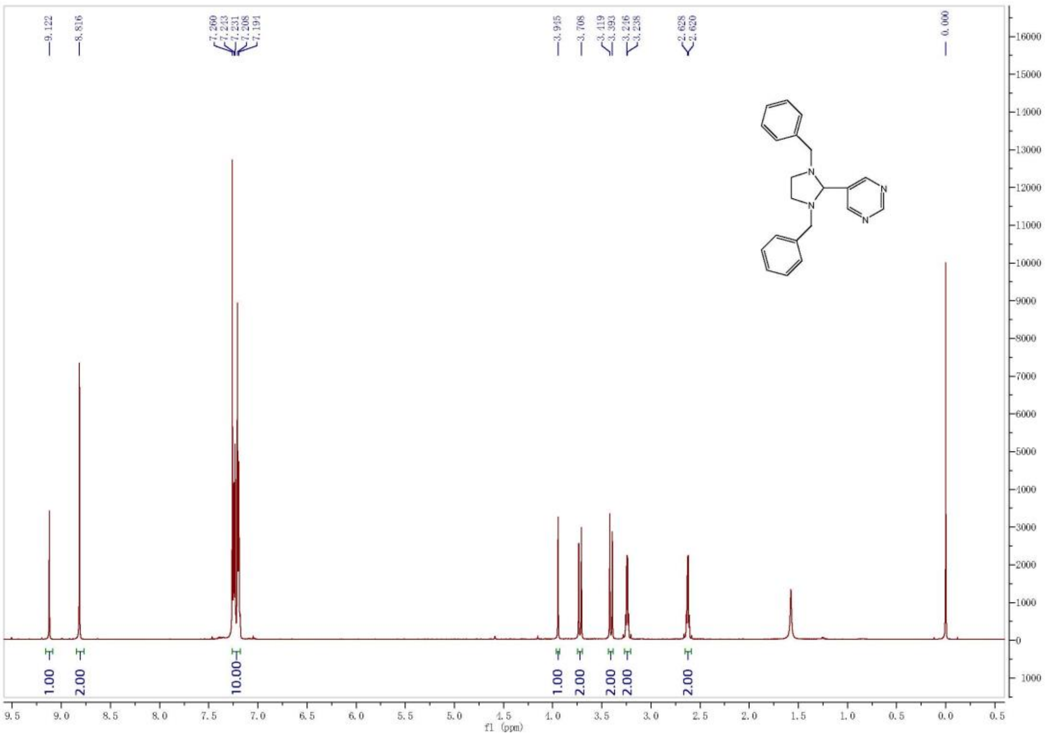

Figure 31 ^{1}H-NMR spectrum of compound **4r**

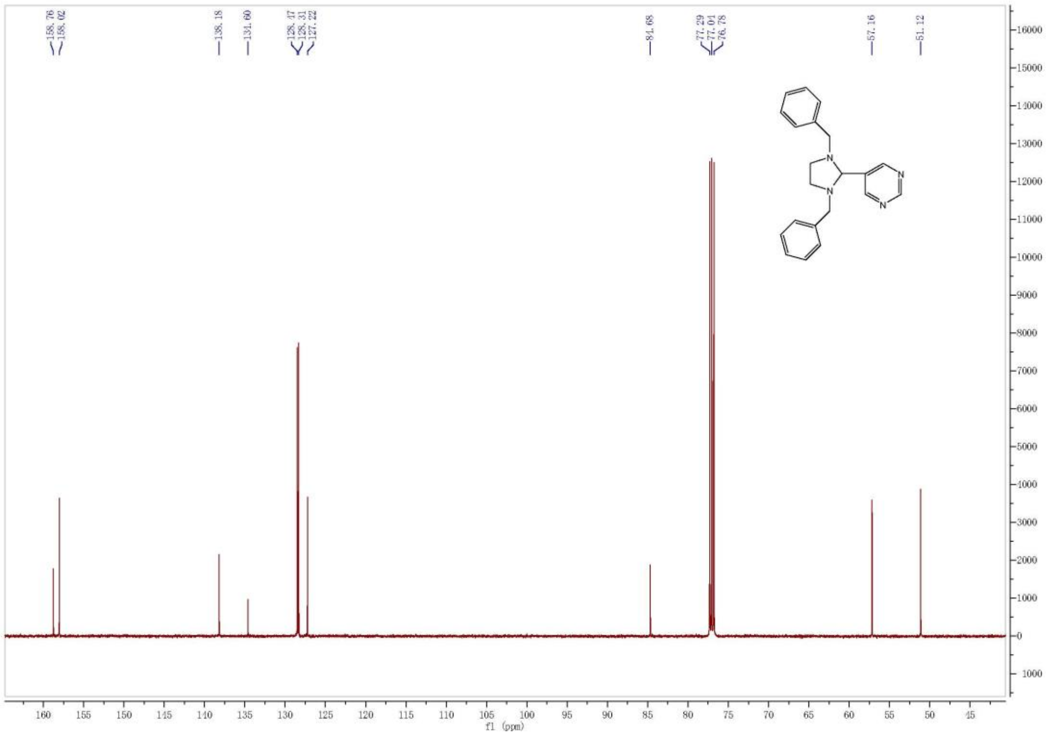

Figure 32 ^{13}C-NMR spectrum of compound **4r**

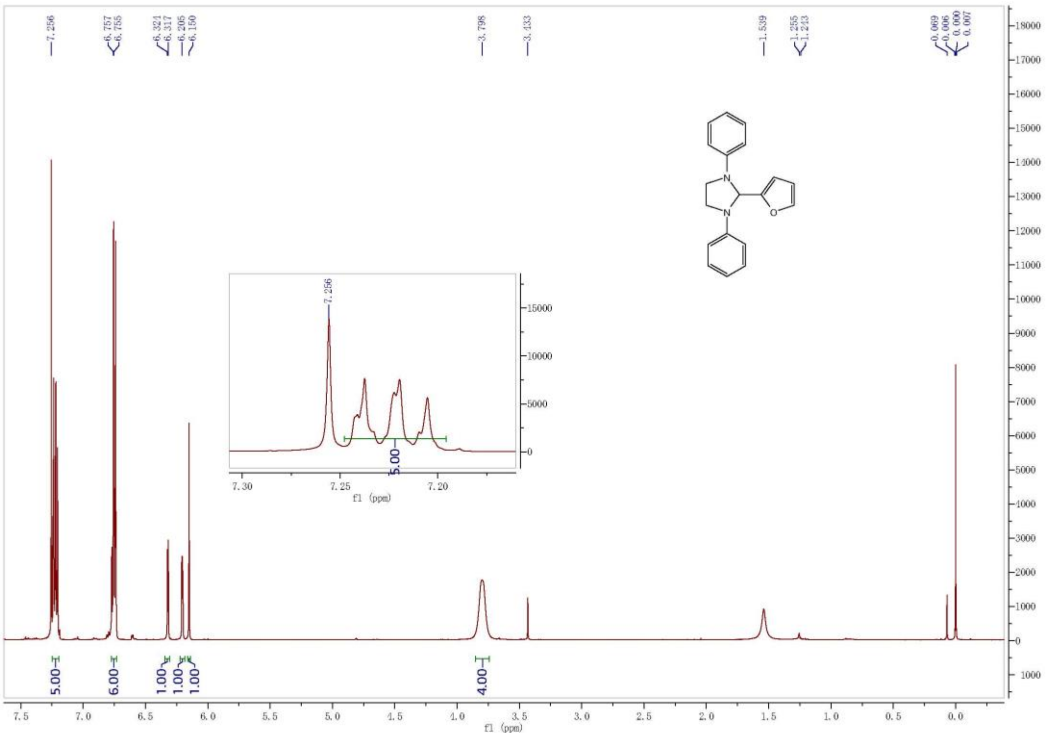

Figure 33 ^{1}H-NMR spectrum of compound **6a**

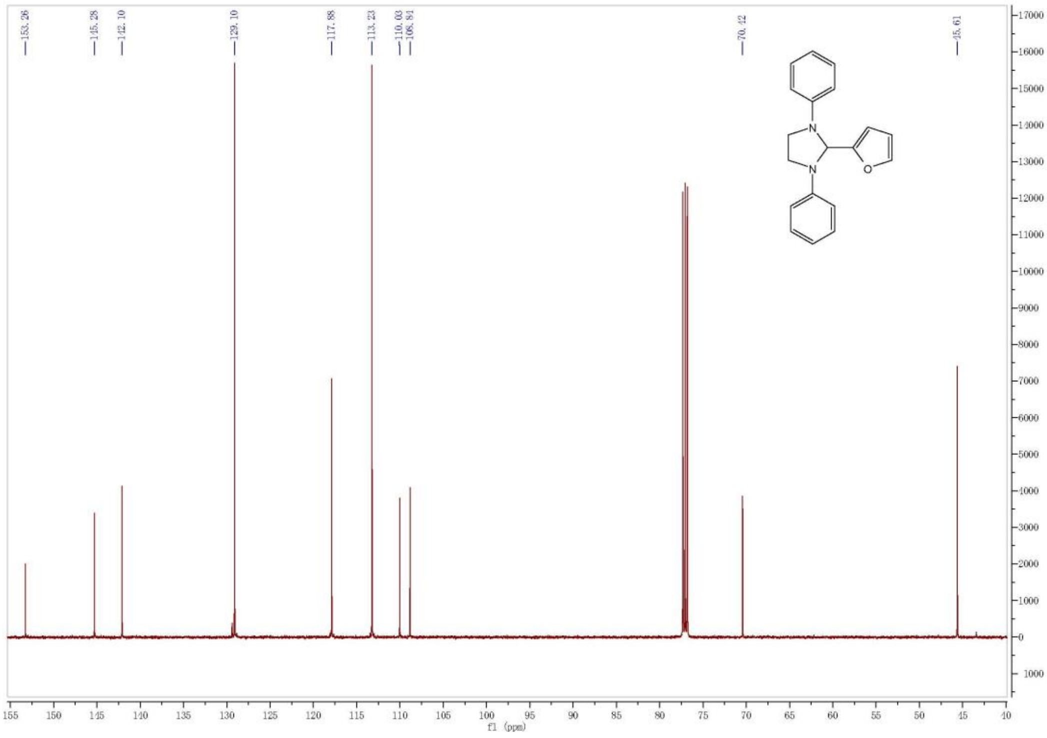

Figure 34 ^{13}C-NMR spectrum of compound **6a**

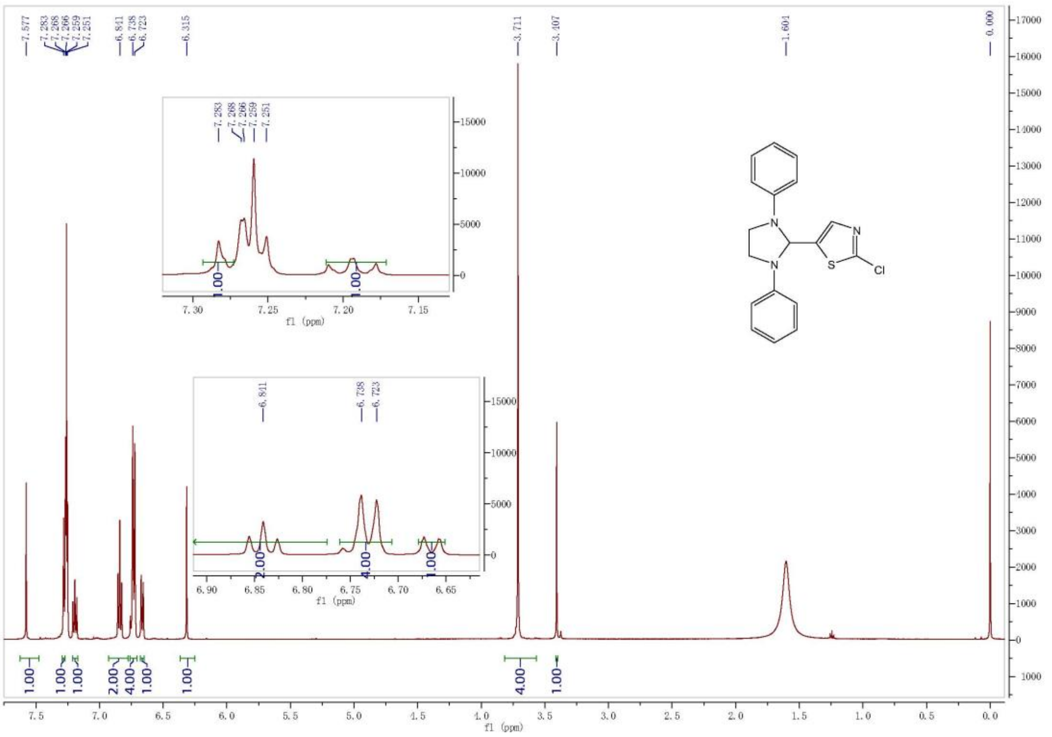

Figure 35 ^{1}H-NMR spectrum of compound **6b**

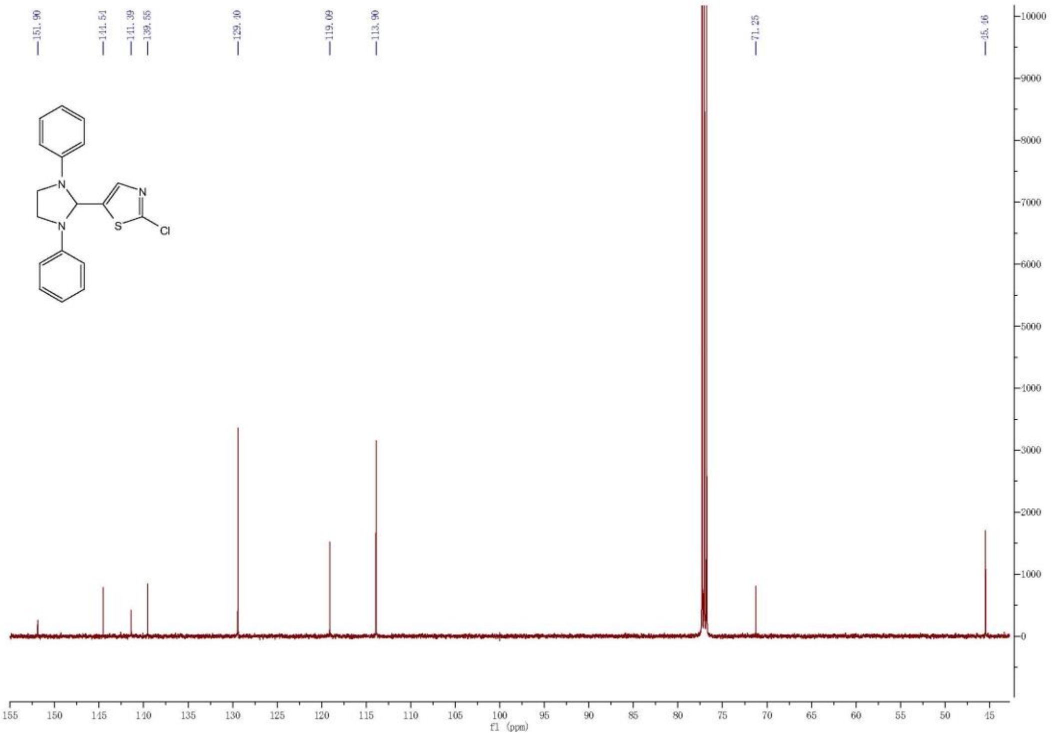

Figure 36 ^{13}C-NMR spectrum of compound **6b**

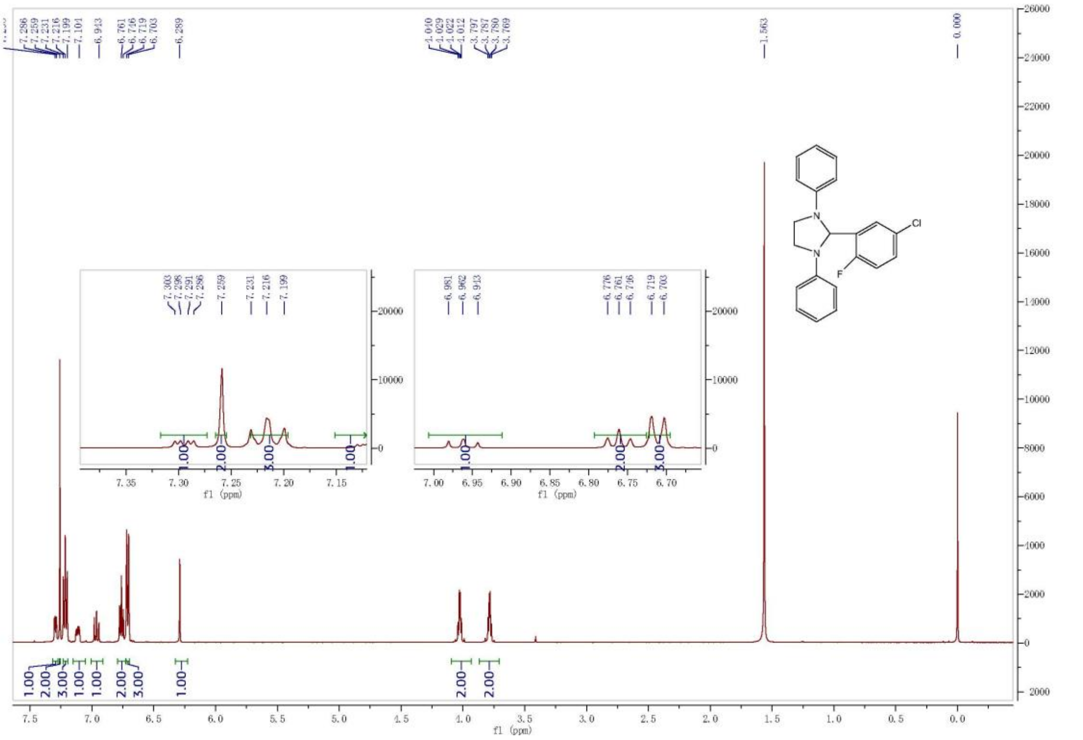

Figure 37 ^{1}H-NMR spectrum of compound **6c**

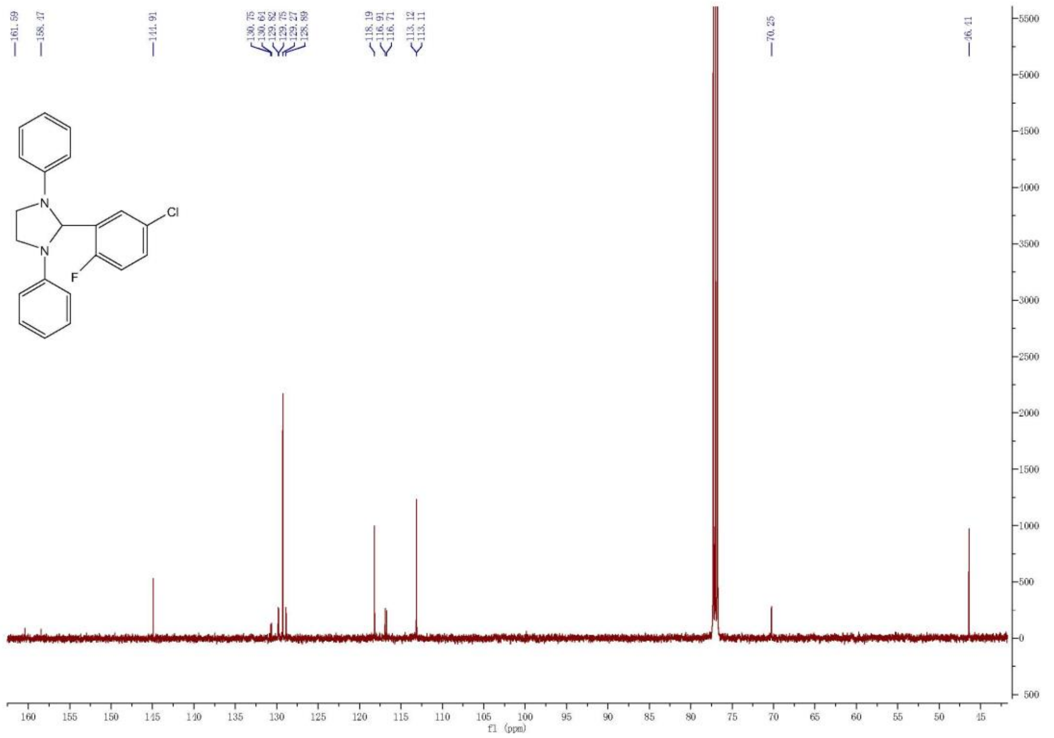

Figure 38 ^{13}C-NMR spectrum of compound **6c**

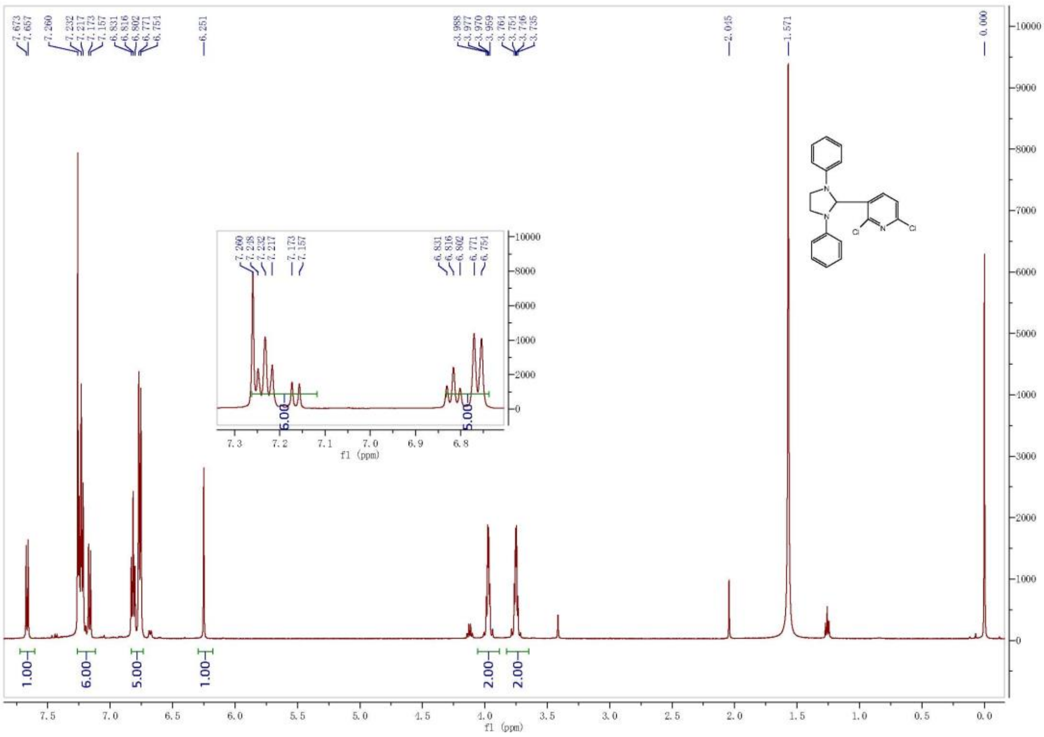

Figure 39 ^{1}H-NMR spectrum of compound **6d**

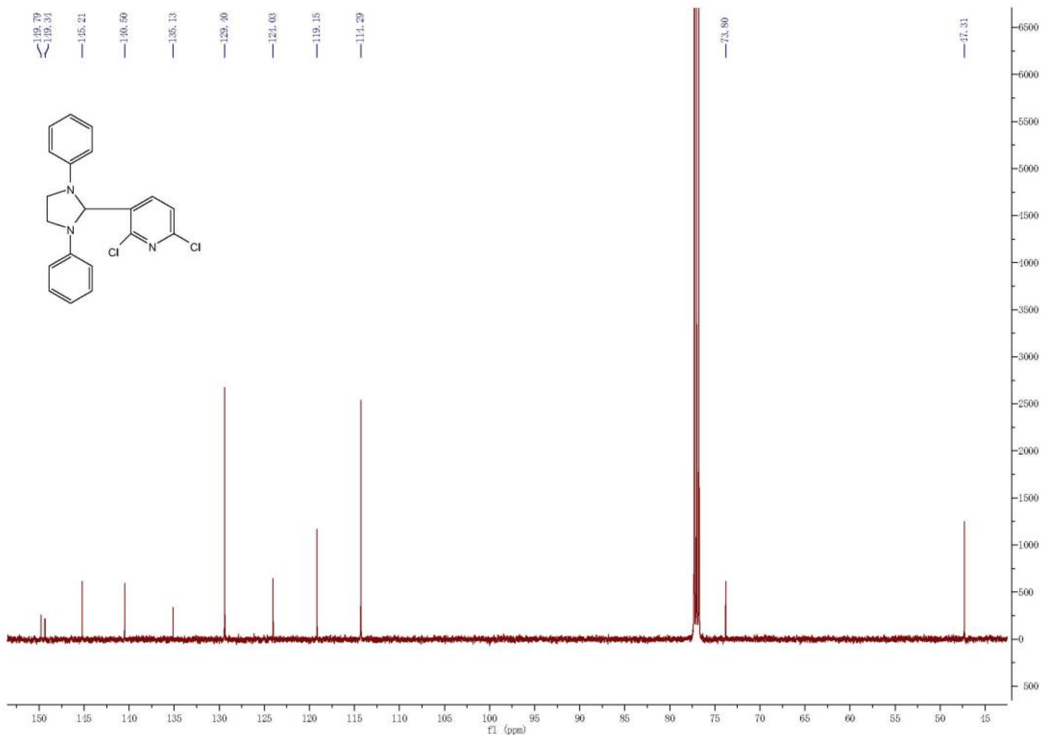

Figure 40 ^{13}C-NMR spectrum of compound **6d**

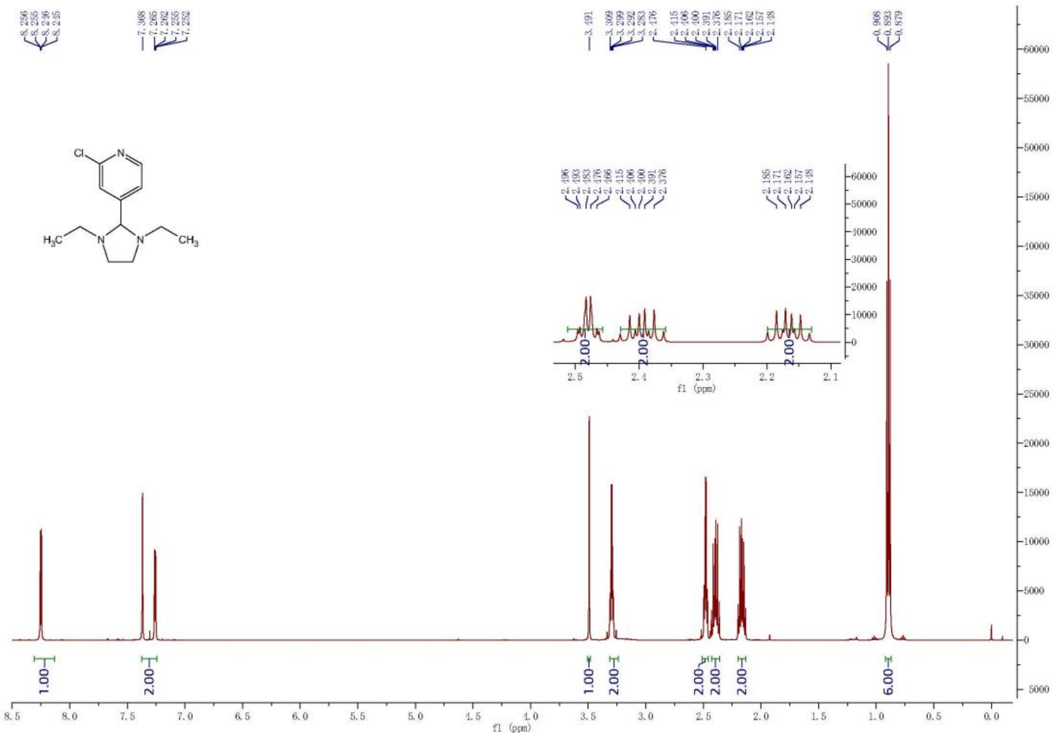

Figure 41 ^{1}H-NMR spectrum of compound **7a**

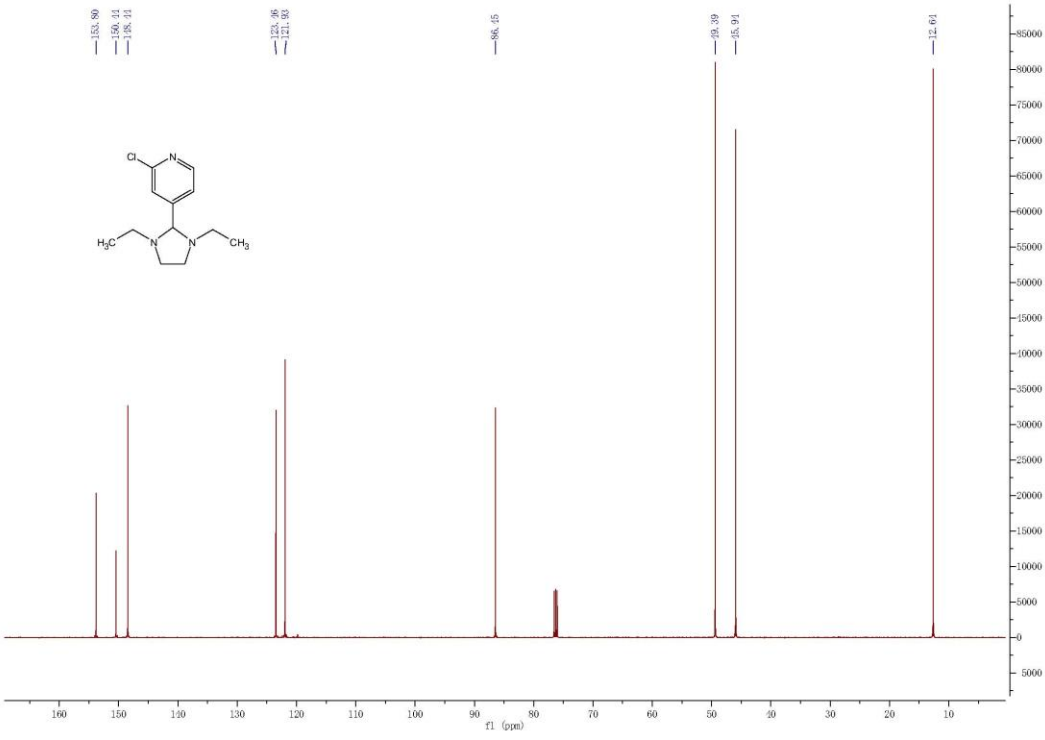

Figure 42 ^{13}C-NMR spectrum of compound **7a**

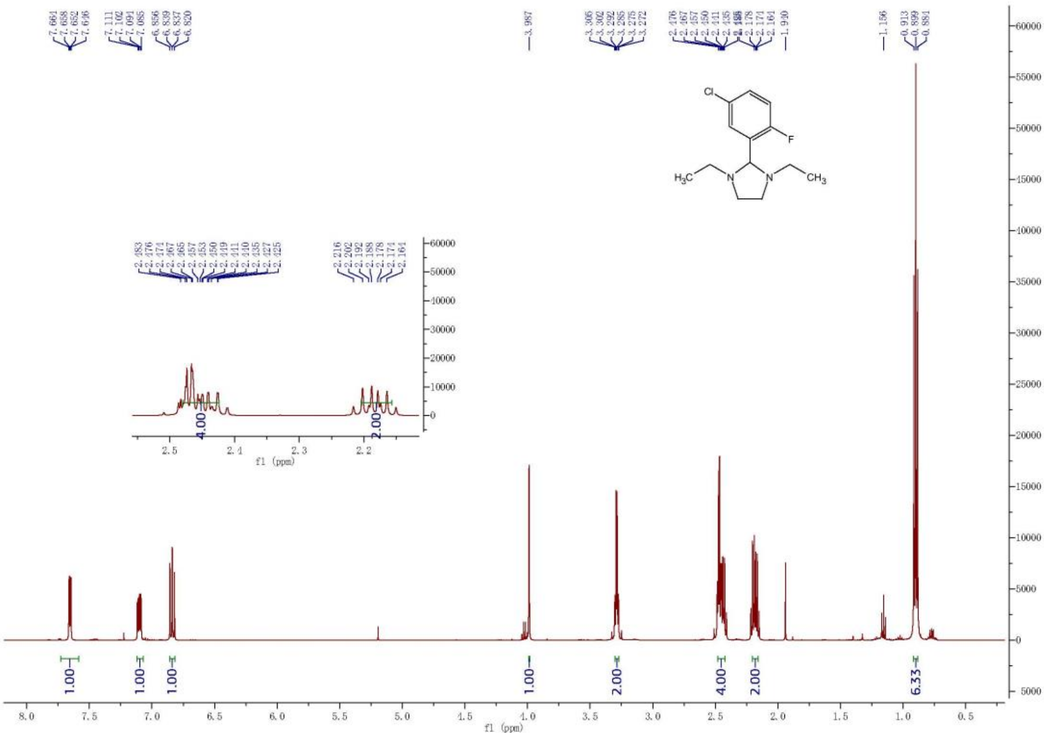

Figure 43 ^{1}H-NMR spectrum of compound **7b**

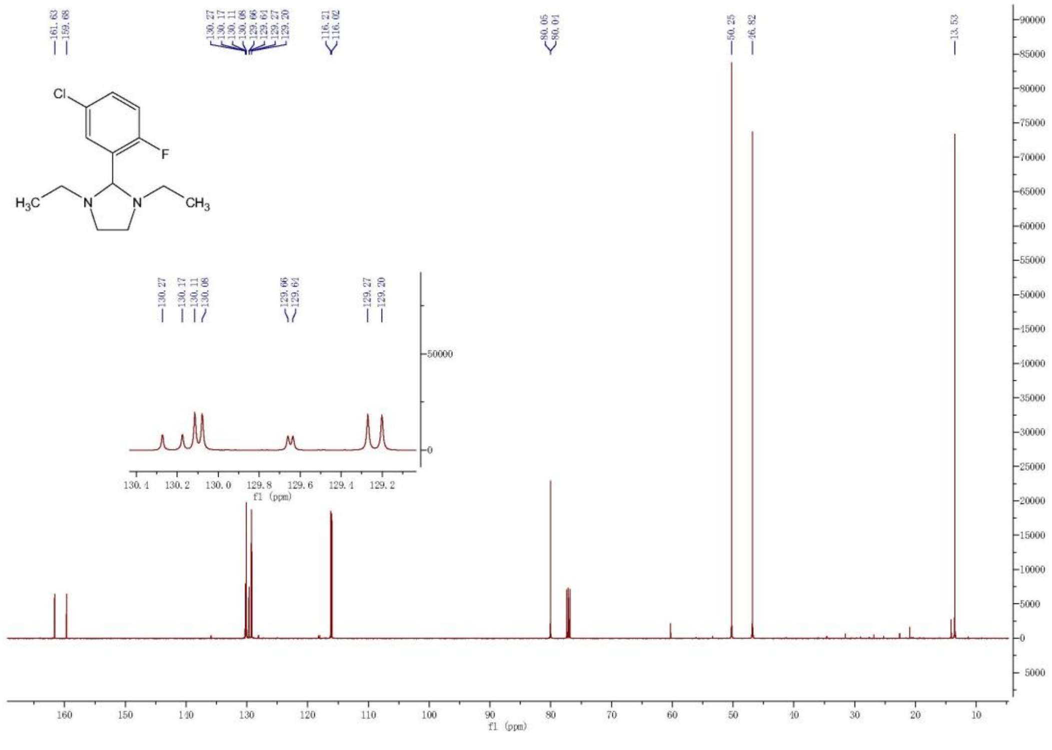

Figure 44 ^{13}C-NMR spectrum of compound **7b**

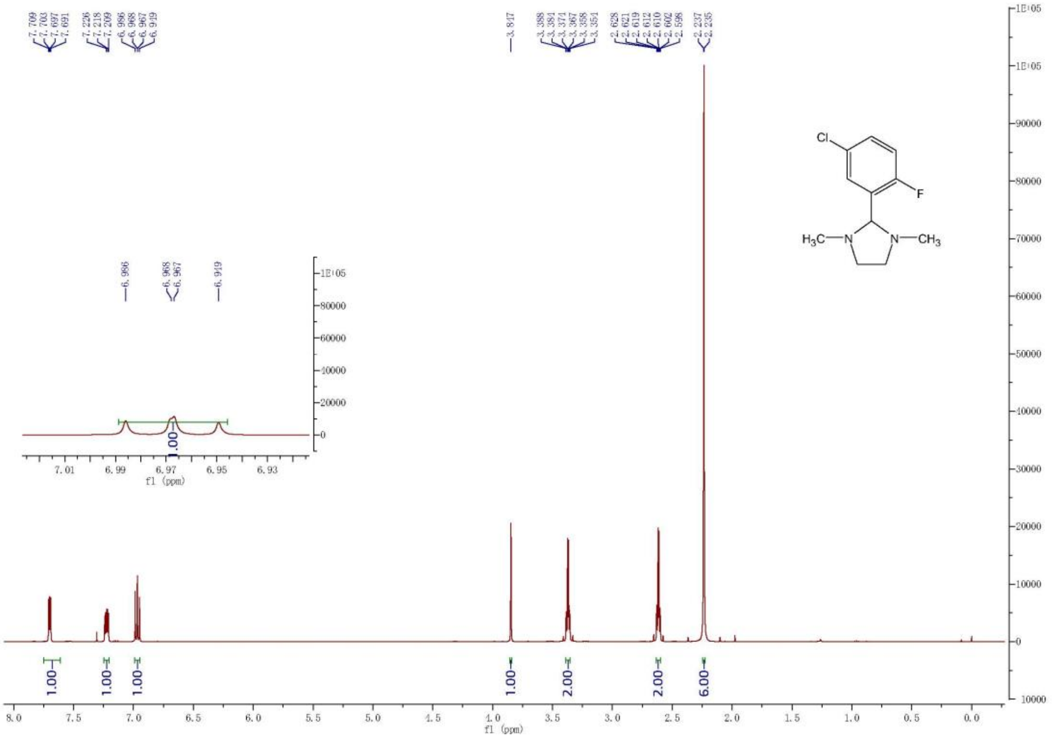

Figure 45 ^{1}H-NMR spectrum of compound **8a**

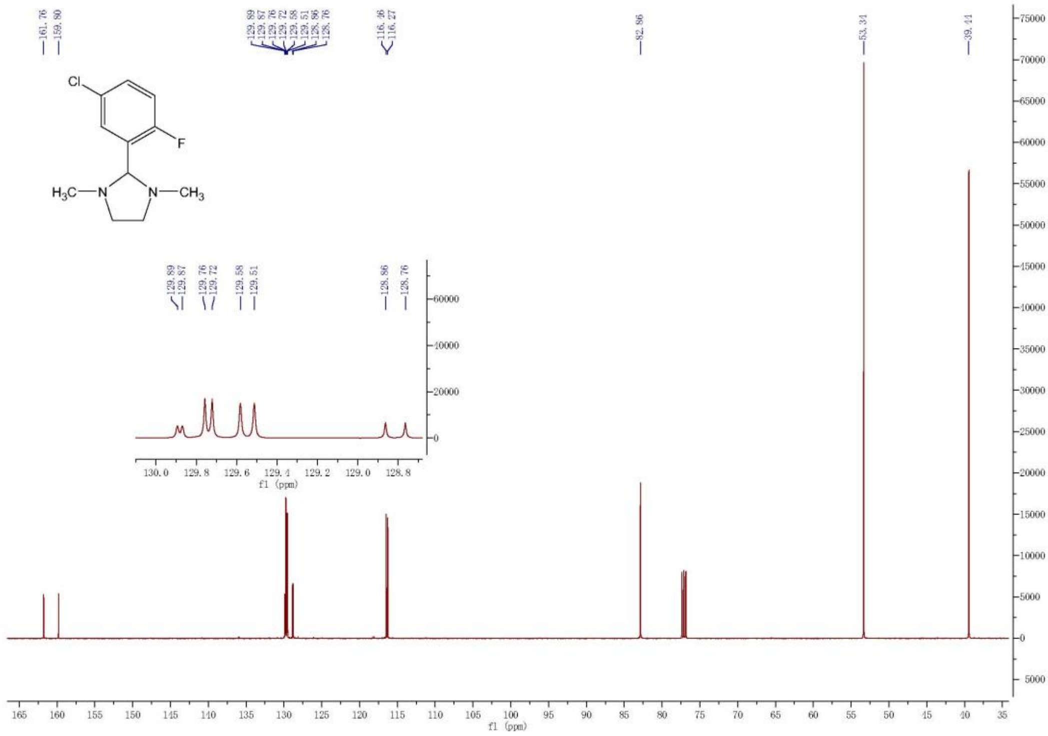

Figure 46 ^{13}C-NMR spectrum of compound **8a**

References and Notes:

1. Jurčík, V.; Wilhelm, R. Preparation of aminals in water. *Tetrahedron* **2004**, *60*, 3205-3210.
2. The spectra of **4p** and **4q** are identical to that provided by SciFinder®.
3. Vanden, E. 2,3-Dichloro.5,6-dicyano-l,4-benzoquinone, a Mild Catalyst for the Formation of Carbon-Nitrogen Bonds. *Tetrahedron* **1995**, *51*, 5813-5818.
